# Rare germline variants in the E-cadherin gene *CDH1* are associated with the risk of brain tumors of neuroepithelial and epithelial origin

**DOI:** 10.1007/s00401-021-02307-1

**Published:** 2021-04-30

**Authors:** Alisa Förster, Frank Brand, Rouzbeh Banan, Robert Hüneburg, Christine A. M. Weber, Wiebke Ewert, Jessica Kronenberg, Christopher Previti, Natalie Elyan, Ulrike Beyer, Helge Martens, Bujung Hong, Jan H. Bräsen, Andreas Erbersdobler, Joachim K. Krauss, Martin Stangel, Amir Samii, Stephan Wolf, Matthias Preller, Stefan Aretz, Bettina Wiese, Christian Hartmann, Ruthild G. Weber

**Affiliations:** 1 Department of Human Genetics OE 6300, Hannover Medical School, Carl-Neuberg-Str. 1, 30625 Hannover, Germany; 2 Department of Neuropathology, Institute of Pathology, Hannover Medical School, Hannover, Germany; 3 Department of Neuropathology, Institute of Pathology, University Hospital Heidelberg, Heidelberg, Germany; 4 National Center for Hereditary Tumor Syndromes, University Hospital Bonn, Bonn, Germany; 5 Department of Internal Medicine I, University Hospital Bonn, Bonn, Germany; 6 Institute for Biophysical Chemistry, Hannover Medical School, Hannover, Germany; 7 Clinical Neuroimmunology and Neurochemistry, Department of Neurology, Hannover Medical School, Hannover, Germany; 8 Center for Systems Neuroscience, University of Veterinary Medicine Hannover, Hannover, Germany; 9 Radiation Biology Department, Institute of Aerospace Medicine, German Aerospace Centre (DLR), Köln, Germany; 10 Genomics and Proteomics Core Facility, High Throughput Sequencing Unit W190, German Cancer Research Center (DKFZ), Heidelberg, Germany; 11 Omics IT and Data Management Core Facility W610, German Cancer Research Center (DKFZ), Heidelberg, Germany; 12 Department of Neurosurgery, Hannover Medical School, Hannover, Germany; 13 Nephropathology, Institute of Pathology, Hannover Medical School, Hannover, Germany; 14 Institute of Pathology, University of Rostock, Rostock, Germany; 15 Department of Neurosurgery, International Neuroscience Institute, Hannover, Germany; 16 Department of Natural Sciences, University of Applied Sciences Bonn-Rhein-Sieg, Rheinbach, Germany; 17 Institute of Human Genetics, Medical Faculty, University of Bonn, Bonn, Germany; 18 Department of Neurology, Henriettenstift, Diakovere Krankenhaus gGmbH, Hannover, Germany

**Keywords:** Familial glioma, Oligodendroglioma, Whole-genome sequencing, *CDH1*, E-cadherin, β-catenin

## Abstract

The genetic basis of brain tumor development is poorly understood. Here, leukocyte DNA of 21 patients from 15 families with ≥ 2 glioma cases each was analyzed by whole-genome or targeted sequencing. As a result, we identified two families with rare germline variants, p.(A592T) or p.(A817V), in the E-cadherin gene *CDH1* that co-segregate with the tumor phenotype, consisting primarily of oligodendrogliomas, WHO grade II/III, IDH-mutant, 1p/19q-codeleted (ODs). Rare *CDH1* variants, previously shown to predispose to gastric and breast cancer, were significantly overrepresented in these glioma families (13.3%) versus controls (1.7%). In 68 individuals from 28 gastric cancer families with pathogenic *CDH1* germline variants, brain tumors, including a pituitary adenoma, were observed in three cases (4.4%), a significantly higher prevalence than in the general population (0.2%). Furthermore, rare *CDH1* variants were identified in tumor DNA of 6/99 (6%) ODs. *CDH1* expression was detected in undifferentiated and differentiating oligodendroglial cells isolated from rat brain. Functional studies using CRISPR/Cas9-mediated knock-in or stably transfected cell models demonstrated that the identified *CDH1* germline variants affect cell membrane expression, cell migration and aggregation. E-cadherin ectodomain containing variant p.(A592T) had an increased intramolecular flexibility in a molecular dynamics simulation model. E-cadherin harboring intracellular variant p.(A817V) showed reduced β-catenin binding resulting in increased cytosolic and nuclear β-catenin levels reverted by treatment with the MAPK interacting serine/threonine kinase 1 inhibitor CGP 57380. Our data provide evidence for a role of deactivating *CDH1* variants in the risk and tumorigenesis of neuroepithelial and epithelial brain tumors, particularly ODs, possibly via WNT/β-catenin signaling.

**Supplementary Information:**

The online version contains supplementary material available at 10.1007/s00401-021-02307-1.

## Introduction

Gliomas, including astrocytomas and oligodendrogliomas, are brain tumors thought to be derived from glial (precursor) cells that originate from the neuroepithelium. In the 2016 World Health Organization (WHO) Classification of Tumors of the Central Nervous System (CNS), gliomas are no longer only defined by histologic characteristics, but also by molecular parameters, e.g. the IDH mutation and 1p/19q codeletion status [37]. The heterogeneous group of diffuse gliomas includes IDH-wildtype or IDH-mutant astrocytomas of WHO grade II or III, IDH-wildtype or IDH-mutant glioblastomas of WHO grade IV, and oligodendrogliomas of WHO grade II or III, which must by definition harbor an IDH mutation and a 1p/19q codeletion, and are thus named (anaplastic) oligodendrogliomas, IDH-mutant and 1p/19q-codeleted (ODs) [37, 51]. Pilocytic astrocytomas of WHO grade I are classified as “other astrocytomas” due to their different histologic and molecular features [37]. Brain tumors also comprise adenomas of anterior pituitary hormone-producing epithelial cells, such as the prolactinoma [36]. To describe tumors with unknown molecular status, the term “not otherwise specified (NOS)” was introduced in the revised WHO classification of 2016, and the diagnosis “oligoastrocytoma” is no longer used [37]. However, a re-classification of gliomas diagnosed prior to 2016 on the basis of the revised classification requires a re-assessment of their molecular genetic status and this, in turn, the availability of tumor tissue.

While most gliomas occur sporadically, familial aggregation, i.e. the occurrence in two or more individuals within a family, was reported in 1999 in approximately 5% of cases [38]. In a subsequent epidemiologic study, first-degree relatives, but not spouses (used as controls), of glioma patients were found to have a significantly increased risk of developing tumors of the same histopathology, indicating a genetic, not an environmental origin of the familial aggregation [39]. Moreover, it is known that certain tumor syndromes, e.g. neurofibromatosis type 1 and 2, tuberous sclerosis complex, familial adenomatous polyposis, and Li-Fraumeni syndrome, are associated with an increased glioma risk, indicating that pathogenic germline variants in the causative genes, i.e. *NF1*, *NF2*, *TSC1*, *TSC2*, *APC*, or *TP53*, predispose to the development of gliomas among other tumors [26, 42]. The underlying disorders are classic monogenic diseases caused by rare variants and are often dominantly inherited.

To identify rare variants and, therefore, novel genes associated with glioma risk, the study of glioma families and the use of next-generation sequencing (NGS) combined with linkage-based data analysis strategies have been instrumental. Using this approach, pathogenic germline variants in the *POT1* gene encoding a member of the telomere shelterin complex were identified as predisposing to glioma, in particular to oligodendroglioma [2]. Making use of a similar strategy, we detected that rare variants in *ADAR* and *RNASEH2B*, two of the genes mutated in Aicardi-Goutières syndrome, a progressive encephalopathy, and a type I interferon signature were associated with glioma risk and tumorigenesis [8]. These recent studies of rare germline variants in glioma families have linked the origin of glioma to different types of cellular pathologies ranging from alterations in telomere biology to inflammation, and, thus, have fundamentally contributed to our understanding of glioma development as a basis for new therapeutic approaches.

In this study, we present data suggesting that heterozygous deactivating variants in the *CDH1* gene increase the risk of brain tumors, primarily of ODs. The *CDH1* gene encodes the cell–cell adhesion protein E-cadherin, the intracellular domain of which binds several catenins, such as β-catenin, and acts as a tumor suppressor [40]. While *CDH1* germline alterations are known to cause specific types of epithelial cancer, e.g. diffuse gastric cancer [20] and invasive lobular breast cancer [63], our data newly add certain neuroepithelial and epithelial brain tumors to the phenotype spectrum caused by rare *CDH1* variants. Moreover, our findings suggest that WNT/β-catenin signaling may be involved downstream of E-cadherin in the development of these tumors, particularly of ODs.

## Materials and methods

### Human samples

The study was approved by the Ethics Boards of Hannover Medical School and the University Hospital Bonn. The glioma family cohort consisted of 15 families, each with ≥ 2 glioma cases, including glioma family 1 from southern Italy and glioma family 2 from northern Germany, recruited in Hannover, Germany. These 15 glioma families comprised 33 patients: 24 (72.7%) with astrocytic tumors, WHO grade I, II, III, or IV, 7 (21.2%) with oligodendroglial tumors, WHO grade II or III, and 2 (6.1%) with subependymomas, WHO grade I. Blood samples of 21 glioma patients were available for genetic testing. The cohort of gastric cancer families, each with a pathogenic *CDH1* germline alteration identified by panel sequencing or multiplex ligation-dependent probe amplification, comprised 68 variant carriers or individuals at risk with unknown carrier status from 28 family, including gastric cancer family 1, 2 and 3, recruited at the National Center for Hereditary Tumor Syndromes, University Hospital Bonn, Germany. Formalin-fixed, paraffin-embedded (FFPE) specimens of oligodendrogliomas were retrieved from the archives of the Department of Neuropathology, Hannover Medical School, Germany. Upon reevaluation by two experienced neuropathologists (RB and CH), 99 of these tumors showed a diffusely infiltrating glial differentiation, an IDH mutation and 1p/19q codeletion, corresponding to oligodendrogliomas, WHO grade II (*n* = 47) or WHO grade III (*n* = 52), IDH-mutant and 1p/19q-codeleted (ODs) according to the 2016 WHO classification. FFPE tumor samples from 65 renal cell carcinomas (RCCs), including 26 chromophobe RCCs (40%), 21 clear cell RCCs (32.3%), and 18 papillary RCCs (27.7%), were retrieved from the archives of the Institute of Pathology, University of Rostock, Germany. Peripheral blood and tumor specimens were subjected to DNA isolation using the QIAamp DNA Blood Maxi Kit (Qiagen, Hilden, Germany) or a standard extraction protocol using phenol–chloroform.

### Whole-genome sequencing (WGS)

WGS was performed on leukocyte DNA of three OD-affected and two unaffected members of glioma family 1 at the German Cancer Research Center, Heidelberg, Germany with a mean coverage of ≥20× using a HiSeq X platform (Illumina, San Diego, CA, USA). Data were processed and aligned to the GRCh37/hg19 reference human genome assembly using the Biomedical Genomics Workbench (version 5.0; Qiagen, Hilden, Germany), and assessed using Ingenuity Variant Analysis (Qiagen) and our in-house NGS data analysis workflow as described in Results and Suppl. Table 1 online resource. Variant minor allele frequencies (MAF) were retrieved from the Genome Aggregation Database (gnomAD) Browser v2.1.1 (http://gnomad.broadinstitute.org/). For prediction of variant deleteriousness, the tools SIFT (http://sift.jcvi.org/), PolyPhen-2 (http://genetics.bwh.harvard.edu/pph2/), and RegulationSpotter (http://www.regulationspotter.org/) were used. Verification of selected non-silent variants detected by WGS (Suppl. Table 2 online resource) was performed using conventional chain termination protocols. Nucleotide numbering of the identified variants reflects cDNA numbering in the NCBI reference sequence (http://www.ncbi.nlm.nih.gov/).

### Targeted sequencing

Targeted sequencing of the entire coding region, including intron–exon boundaries, of *CDH1* (NM_004360) was performed on leukocyte DNA of 18 patients from 14 glioma families not analyzed by WGS, and tumor DNA of 99 ODs. Mutational analysis of *CDH1* exons 14 to 16 was done on tumor DNA of 65 RCCs. Amplicons generated using customized oligonucleotides (Suppl. Table 3 online resource) and standard molecular techniques were sequenced by means of conventional chain termination protocols on a 3130xl Genetic Analyzer (Thermo Fisher Scientific, Waltham, MA, USA). Leukocyte DNA of the 18 patients from 14 glioma families not analyzed by WGS was additionally screened for variants of the entire coding region of *POT1* (NM_015450; oligonucleotides listed in Suppl. Table 4 online resource). All non-silent variants were assessed with respect to MAF and pathogenicity, as described above.

### Isolation of rat oligodendroglial cells

Primary oligodendroglial cultures were prepared from neonatal Sprague–Dawley rats (P0–P3) as described previously [16]. Details are given in Supplementary material online resource. Purity of cultures was routinely > 90%, as determined by double staining for GFAP to detect astrocyte contamination and OX-42 for microglia.

### Cloning of expression constructs and site-directed mutagenesis

The full-length *CDH1* open reading frame was amplified from hE-cadherin-pcDNA3 (#45769, Addgene, Watertown, MA, USA) and subcloned into the *Xh*oI-linearized pIRES-EGFP-puro vector (#45567, Addgene) using customized oligonucleotides (Suppl. Table 5 online resource) and the In-Fusion HD Cloning Kit (Takara Bio, Kusatsu, Japan). The *CDH1* variants, c.1774G > A p.(A592T) and c.2450C > T p.(A817V), were inserted into the resulting *CDH1* expression construct using the Phusion Site-Directed Mutagenesis Kit (Thermo Fisher Scientific) and customized oligonucleotides (Suppl. Table 6 online resource). The sequences of all generated constructs, i.e. pIRES-EGFP-puro (vector control), pIRES-CDH1 wildtype-EGFP-puro (WT), pIRES-CDH1 A592T-EGFP-puro (A592T), and pIRES-CDH1 A817V-EGFP-puro (A817V), were verified using restriction enzyme digest and direct sequencing.

### Cell culture, transfection, and treatment

HEK293T cells were cultured in high-glucose Dulbecco’s Modified Eagle Medium (DMEM, Merck, Darmstadt, Germany), and CHO-K1 (CHO) cells in DMEM/F-12 (Merck). Both cell culture media were supplemented with 10% fetal bovine serum, 2 mM l-glutamine, and 1% penicillin/streptomycin (all Thermo Fisher Scientific). Transfection of generated *CDH1* expression constructs was done using the Lipofectamine 3000 reagent (Thermo Fisher Scientific). For stable expression, transfected CHO cells were treated with 20 µg/ml puromycin (Thermo Fisher Scientific) for 21 days. GFP-positive cells were isolated using a MoFlo XDP cell sorter (Beckman-Coulter, Brea, MA, USA). To inhibit MAPK interacting serine/threonine kinase 1 (MNK1), HEK293T and CHO cells were treated with 75 or 200 µM CGP 57380 (Cayman Chemicals, Ann Arbor, MI, USA) for 24 or 72 h.

### Immunofluorescence microscopy

To investigate E-cadherin, oligodendrocyte transcription factor 2 (Olig2) and myelin basic protein (MBP) expression in rat oligodendroglial (precursor) cells, cells were fixed after 4 days in either proliferation or differentiation medium with 4% paraformaldehyde (PFA) for 15 min at 4 °C. Cells were permeabilized with 0.25% Triton X-100 in phosphate-buffered saline (PBS) for 25 min and treated with 6% bovine serum albumin (BSA), 0.1% Triton X-100 in PBS for 1 h. The primary antibodies given in Supplementary material online resource were diluted in 1% BSA, 0.1% Triton X-100 in PBS and incubated at 4 °C overnight. Goat anti-mouse or anti-rabbit Alexa Fluor 488- or 568-conjugated secondary antibodies (all Thermo Fisher Scientific; dilution 1:500) were incubated for 1 h at room temperature (RT). Cells were counterstained with 4′,6-diamidino-2-phenylindole (DAPI) and embedded in Mowiol mounting medium. Images were captured using a DM IRB confocal microscope (Leica Microsystems, Wetzlar, Germany). To analyze E-cadherin expression in stably transfected CHO cells, cells were fixed with 4% PFA in PBS. Rabbit anti-E-cadherin (#3195, Cell Signaling Technology (CST), Danvers, MA, USA; dilution 1:2000) was used as primary and Alexa Fluor 568-conjugated goat anti-rabbit (Thermo Fisher Scientific; dilution 1:500) as secondary antibody. Cells were counterstained with DAPI. Images were acquired using a LSM980 confocal microscope (Zeiss, Oberkochen, Germany).

### Quantitative and qualitative mRNA expression analysis

To determine *Cdh1* mRNA expression in rat oligodendroglial (precursor) cells, RNA was isolated from cells cultured in proliferation or differentiation medium for 4 or 6 days using the RNeasy Mini Kit (Qiagen). cDNA was synthesized using the Superscript III First-Strand Synthesis System (Thermo Fisher Scientific). For quantitative *Cdh1* mRNA analysis, the PowerUp SYBR Green Master Mix (Thermo Fisher Scientific) was used with specific primers for rat *Cdh1* cDNA (NM_031334) and rat *Pgk1* cDNA (NM_053291) as control (Suppl. Tables 7 and 8 online resource). Each sample was normalized to *Pgk1*, and comparative C_t_ quantification was applied. For qualitative *Cdh1* mRNA analysis, exon-spanning oligonucleotides specific for rat *Cdh1* cDNA were used for PCR amplification (Suppl. Table 9 online resource). Generated amplicons were analyzed by direct sequencing.

### Immunohistochemistry

To determine E-cadherin expression in *CDH1* wildtype ODs, WHO grade II/III, immunostaining of 3 µm thick FFPE sections of 19 ODs was done after heat-induced epitope retrieval in 10 mM citrate buffer pH 6.0. Using rabbit anti-E-cadherin (#24E10, CST; dilution 1:50) as primary antibody, slides were incubated at 4 °C overnight. As secondary antibody, horseradish-peroxidase-labeled goat anti-rabbit (#A24537, Thermo Fisher Scientific; dilution 1:200) was used. Sections were counterstained with Mayer’s hemalum solution. For histological evaluation, consecutive sections of all specimens were hematoxylin–eosin stained. Images were acquired using a BX46 microscope and a XC50 camera (all Olympus, Shinjuku, Japan).

### CRISPR/Cas9-mediated knock-in of *CDH1*:c.2450C > T p.(A817V)

The knock-in of *CDH1*:c.2450C > T p.(A817V) in HEK293T cells was done using a protocol for CRISPR/Cas9-mediated RNA-guided genome editing [49]. A guide RNA (gRNA) sequence targeting *CDH1* exon 16 was designed using the CRISPOR web-based tool (http://www.crispor.tefor.net), along with sense and antisense oligonucleotides, and a 200 bp-oligonucleotide containing the *CDH1*:c.2450C > T p.(A817V) variant and a synonymous PAM site variant to prevent further Cas9 cleavage as a homology-directed repair (HDR) template (Suppl. Table 10 online resource). Details are given in Supplementary material online resource. Coding gRNA off-target sites were analyzed by direct sequencing (primer sequences are listed in Suppl. Table 11 online resource) in the three HEK293T cell clones selected for further analysis harboring either *CDH1* wildtype (WT/WT), a heterozygous knock-in (WT/A817V) or a homozygous knock-in (A817V/A817V).

### Cell fractionation

Cytosolic and nuclear fractions of HEK293T E-cadherin A817V knock-in and control cell clones were generated using the NE-PER Nuclear and Cytoplasmic Extraction Reagent Kit (Thermo Fisher Scientific). Protein concentrations were determined using the Pierce BCA Protein Assay Kit (Thermo Fisher Scientific).

### Immunoprecipitation

To analyze binding of wildtype and mutant E-cadherin to β-catenin by immunoprecipitation (IP), E-cadherin A817V knock-in and control cell clones (8.0 × 10^6^) were seeded in Petri dishes. Cells were washed with ice-cold PBS and lysed for 30 min on ice in 0.5 ml IP lysis buffer (20 mM Tris–HCl pH 8.0, 50 mM sodium fluoride, 1 mM sodium orthovanadate, 1% Nonidet P40, protease and phosphatase inhibitors (Roche, Basel, Switzerland)). After adding 1.5 µl anti-β-catenin antibody (#2698, L87A12, CST), lysates were rotated overnight at 4 °C. Protein G Sepharose beads (GE, Boston, MA, USA) were equilibrated in IP lysis buffer and incubated with the lysates for 4 h at 4 °C. After washing 5× with IP lysis buffer, protein eluted from the beads using Laemmli buffer (62.5 mM Tris–HCl pH 6.8, 10% glycerol, 2% SDS, 5% 2-mercaptoethanol, 1 mM EDTA, 0.01% bromophenol blue) was detected by Western blot analysis.

### Western blot analysis

After sodium dodecylsulfate-polyacrylamide gel electrophoresis and semidry electroblotting, polyvinylidene difluoride or nitrocellulose membranes (GE) were treated with 5% fat-free milk powder in PBS with 0.05% Tween 20 (PBST) as blocking agent. The primary antibodies given in Supplementary material online resource were diluted in 5% (w/v) BSA in PBST and used for immunodetection. After incubation overnight at 4 °C, membranes were exposed to the appropriate horseradish peroxidase-conjugated secondary antibody (Thermo Fisher Scientific; dilution 1:3,000) in 5% fat-free milk powder in PBST for 90 min at RT, and developed using the Pierce SuperSignal West Atto or Dura Substrate detection kit (Thermo Fisher Scientific). Signals were acquired using the Fusion FX7 gel documentation system (Vilber, Collégien, France). Densitometric quantification of protein bands was performed using Fiji software [54].

### Slow aggregation assay

To assess aggregation of HEK293T A817V knock-in clones and CHO cells stably expressing wildtype or mutant E-cadherin, single cell suspensions were seeded in triplicate at a density of 4 × 10^4^ cells/well in 96-well plates coated with 50 µl/well of a 0.7% agar solution [10]. Cell aggregation was documented after 72 h (HEK293T cell clones) or 24 h (CHO cells) using Leica DM IL LED inverted microscope and MC190 HD camera (all Leica Microsystems).

### Wound healing assay

To analyze cell migration of HEK293T A817V knock-in clones and CHO cells expressing wildtype or mutant E-cadherin, 2.4 × 10^5^ HEK293T or 1 × 10^5^ CHO cells/well were seeded in 96-well plates and treated with 30 µg/ml mitomycin C (Santa Cruz Biotechnology, Dallas, TX, USA) for 3 h to inhibit cell proliferation. Scratches were applied followed by washing twice to remove detached cells and mitomycin C. Cell-free areas were documented at 0 and 16 h (CHO cells) or 0 and 24 h (HEK293T cell clones) after applying scratches using Leica DM IL LED inverted microscope and MC190 HD camera (all Leica Microsystems). For each documented image, the area of the cell-free gap was measured using the Fiji software, and within each experiment, mean values per cell line were calculated from at least three images. Cell migration was determined as the ratio of the difference between gap areas at 0 and 16 h (CHO cells) or 0 and 24 h (HEK293T cell clones) to the gap area at 0 h. For each cell line, mean values were calculated from three or four independent experiments, and cell migration relative to vector control cells (CHO cells) or WT/WT cells (HEK293T cell clones) was determined.

### Cell viability assay

To assess the viability of HEK293T A817V knock-in clones, 2 × 10^4^ cells/well were seeded in 96-well plates. Cell viability was determined 44 h after seeding using the CellTiter 96® AQueous One Solution Cell Proliferation Assay (MTS assay; Promega, Madison, WI, USA). Absorbance at 490 nm was measured using a FLUOstar Omega microplate reader (BMG Labtech, Ortenberg, Germany). To investigate the effect of the MNK1 inhibitor CGP 57380 on the viability of HEK293T cell clones, 1 × 10^4^ cells/well were seeded in 96-well plates. CGP 57380 (200 µM) was applied 20 h after seeding and left to incubate for 72 h before an MTS assay was performed.

### Flow cytometry

To compare cell membrane expression of wildtype and mutant E-cadherin, stably transfected CHO cells were seeded on culture plates, grown until confluency, and harvested by scraping from plates. Subsequently, 2.0 × 10^6^ cells were resuspended in 1% PFA in PBS and incubated for 1 h on ice. After washing twice in PBS, cells were incubated in 70% ice-cold ethanol at 4 °C overnight. Again, cells were washed twice in PBS, resuspended in fluorescence-activated cell sorting (FACS) buffer (0.5% (w/v) BSA, 1 mM EDTA in PBS) and incubated with rabbit anti-E-cadherin (#3195, 24E10, CST; 1 µl/10^6^ cells) for 1 h at RT. After washing thrice in PBS, cells were incubated with goat anti-rabbit Alexa Fluor 647-conjugated secondary antibody (#A27040, Thermo Fisher Scientific) for 30 min at RT. Again, cells were washed thrice in PBS, resuspended in FACS buffer, and their fluorescence intensities were measured using a FACSCanto II cytometer (Becton, Dickinson and Company, Franklin Lakes, NJ, USA). FCS-files were analyzed using Flowing Software 2.5.1 (Perttu Terho, University of Turku, Finland).

### Molecular dynamics simulations

Coordinates of the crystal structure of the mature ectodomain comprising extracellular cadherin (EC) domains EC1 to EC5 of murine E-cadherin (Protein Data Bank (PDB) code: 3Q2V [23]) were obtained from the PDB database [5]. A crystal structure of the human ectodomain is only available of EC1 and EC2, and the sequence identity of the murine and human E-cadherin ectodomains is 82%. The human A592T variant (position in the pre-protein) was introduced at the corresponding conserved position 438 (A592/438T) into the crystallized sequence of the mature murine protein using the Schrödinger Suite (Schrödinger Release 2019-3), and both wildtype and mutant E-cadherin were energy-minimized using MacroModel and the OPLS3 force field [22]. All molecular dynamics (MD) simulations were conducted using NAMD 2.13 [45] and the CHARMM36 force field [6], as described in Supplementary material online resource.

### Statistical analysis

Data are presented as mean ± standard deviation (SD). Statistical significance was calculated using two-tailed Student’s *t* or Fisher’s exact test, whereby *p* values of ≤ 0.05 (*), ≤ 0.01 (**), and ≤ 0.001 (***) were considered statistically significant.

## Results

### Rare variants in the tumor suppressor gene *CDH1* are associated with glioma risk and tumorigenesis

**Fig. 1:**
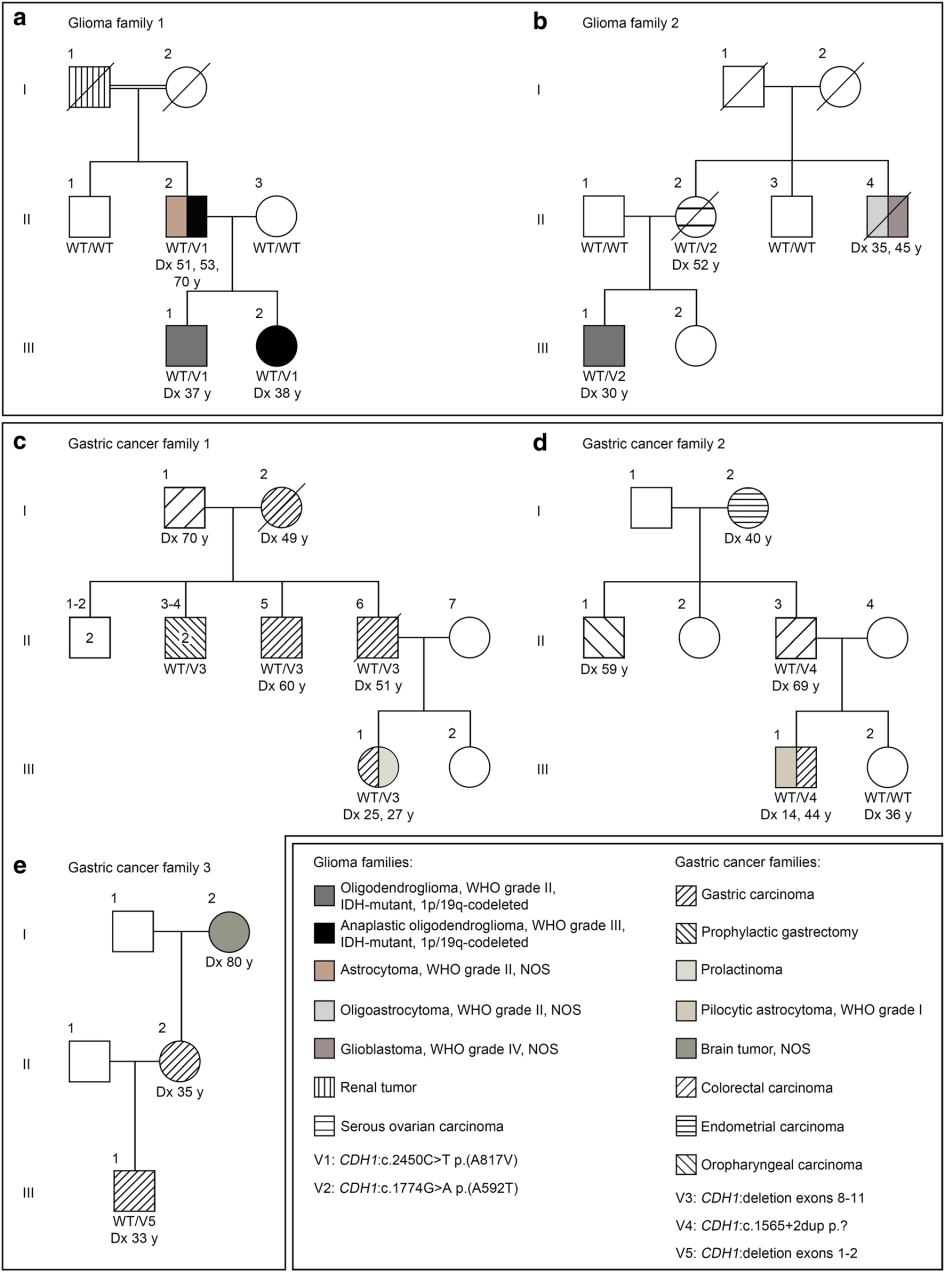
*CDH1* germline variants co-segregate with the tumor phenotype in glioma families (**a, b**), and gastric cancer families with pathogenic *CDH1* variants also manifest brain tumors (**c**–**e**). **a** Pedigree of glioma family 1 and segregation of the rare germline variant V1: *CDH1*:c.2450C > T p.(A817V) detected by WGS. Patients III.1 and III.2 as well as their father II.2, each diagnosed with ODs, WHO grade II/III (recurrent tumors of an astrocytoma, WHO grade II, NOS in patient II.2), were heterozygous variant carriers, while the tumor-unaffected family members II.1 and II.3 were non-carriers. **b** Pedigree of glioma family 2 and segregation of the rare germline variant V2: *CDH1*:c.1774G > A p.(A592T) detected by targeted *CDH1* sequencing. Patient III.1 diagnosed with an OD, WHO grade II and his mother II.2 affected by a serous ovarian carcinoma were heterozygous variant carriers, while the tumor-unaffected family members II.1 and II.3 were non-carriers. **c** Pedigree of gastric cancer family 1 and segregation of the germline alteration V3: deletion of *CDH1* exons 8 to 11. Patients II.5, II.6, and III.1 were heterozygous carriers of the *CDH1* deletion and suffered from gastric cancer. Individuals II.3 and II.4 who were also heterozygous deletion carriers had prophylactic gastrectomy. Patient III.1 additionally had a pituitary adenoma, i.e. a prolactinoma. **d** Pedigree of gastric cancer family 2 and segregation of the germline variant V4: *CDH1*:c.1565 + 2dup p.?. Patients II.3 and III.1 diagnosed with colorectal or gastric cancer, respectively, were heterozygous variant carriers, while tumor-unaffected individual III.2 was a non-carrier. Patient III.1 was additionally diagnosed with a pilocytic astrocytoma, WHO grade I. **e** Pedigree of gastric cancer family 3 and segregation of the germline alteration V5: deletion of *CDH1* exons 1 to 2. Patient III.1 diagnosed with gastric cancer was a heterozygous carrier of the deletion, while DNA of his mother II.2 with gastric cancer and his maternal grandmother I.2 with a brain tumor, NOS was unavailable for genetic testing. *WT* wildtype; *V* variant; *Dx* age at diagnosis; *y* years; *NOS* not otherwise specified

In glioma family 1, four individuals in three consecutive generations were either affected by a glioma, i.e. an OD, WHO grade II in patient III.1, an anaplastic OD, WHO grade III in patient III.2, and an astrocytoma, WHO grade II, NOS recurring twice as anaplastic OD, WHO grade III after two and 19 years in patient II.2, or a renal tumor (patient I.1) (Fig. 1a). To identify germline variants associated with glioma risk, WGS was conducted on leukocyte DNA of patients II.2, III.1, III.2, and their tumor-unaffected relatives II.1 and II.3, and a linkage-based strategy was used for data analysis (Suppl. Table 1 online resource). Variants with a defined quality score (read depth ≥ 10, call quality ≥ 50, allele fraction ≥ 20%) shared by the three glioma-affected individuals and not present in two unaffected relatives were prioritized. Of these 111,708 variants, 30 were rare (MAF ≤ 0.5%), non-silent (i.e. non-synonymous missense, nonsense, frameshift, in-frame indels, and splice site variants) or located in promoter regions, predicted to be deleterious by at least one prediction tool (i.e. SIFT, PolyPhen-2 or RegulationSpotter), and verified by direct sequencing in the case of non-silent variants (Suppl. Table 2 online resource). One of these 30 variants, the heterozygous *CDH1*:c.2450C > T p.(A817V) variant, was located in a cancer predisposing gene [48], and co-segregated with the glioma phenotype in glioma family 1, as verified by Sanger sequencing (Suppl. Figure 1 online resource). By targeted sequencing of *CDH1* on leukocyte DNA of additional glioma families (*n* = 14), we identified another rare non-silent *CDH1* variant that was predicted to be deleterious, *CDH1*:c.1774G > A p.(A592T), in one family, glioma family 2. In addition to the two gliomas, i.e. an OD, WHO grade II in patient III.1 and an oligoastrocytoma, WHO grade II, NOS recurring 10 years later as glioblastoma, WHO grade IV, NOS in patient II.4 (both tumors could not be characterized further because material was no longer available), occurring in glioma family 2, a serous ovarian carcinoma was diagnosed in patient II.2 (Fig. 1b). Segregation analysis revealed that only the tumor-affected individuals of whom DNA was available (patients III.1 and II.2) were carriers of the *CDH1* variant, while their unaffected family members (relatives II.1 and II.3) were not (Suppl. Fig. 1 online resource). Taken together, the frequency of rare non-silent *CDH1* germline variants predicted to be deleterious was significantly increased in our cohort of glioma families (2/15, 13.3%) compared to controls from the gnomAD v2.1.1 dataset (1.7%) (Table 1). No rare deleterious variants in the *POT1* gene [2] were detected in the 15 glioma families.

**Table 1:** Number of carriers of rare non-silent *CDH1* germline variants predicted to be deleterious in our cohort of glioma families compared to controls

	Glioma families	Controls	*p* value
*CDH1* variant			
Carriers	2 (13.3%)	1031 (1.7%)	0.0267^*^
Non-carriers	13	59,115	
Total	15	60,146	

Given are all carriers and non-carriers of rare (MAF ≤ 0.5%) non-silent *CDH1* germline variants that are predicted to be deleterious by one of two prediction tools (SIFT or PolyPhen-2 according to the gnomAD database) in our glioma families compared to gnomAD dataset v2.1.1 (controls) (http://gnomad.broadinstitute.org/)

*p ≤ 0.05 (two-tailed Fisher’s exact test)

Next, we determined the prevalence of brain tumors in gastric cancer families carrying pathogenic *CDH1* germline variants. Brain tumors were observed in three of 28 (10.7%) gastric cancer families with pathogenic *CDH1* variants. Patient III.1 of gastric cancer family 1 with a heterozygous deletion of *CDH1* exons 8 to 11 was diagnosed with a pituitary adenoma, i.e. a prolactinoma, at age 27 years after having had a gastric carcinoma two years earlier (Fig. 1c). Patient III.1 of gastric cancer family 2 with the heterozygous *CDH1*:c.1565 + 2dup p.? variant was diagnosed with a pilocytic astrocytoma, WHO grade I at age 14 years and a gastric carcinoma at 44 years (Fig. 1d). In gastric cancer family 3, the maternal grandmother (I.2) of patient III.1, who carried a heterozygous deletion of *CDH1* exons 1 to 2 and was affected by gastric cancer, suffered from a brain tumor, NOS (Fig. 1e). As brain tumors occurred in three of 68 individuals from gastric cancer families with pathogenic *CDH1* variants, the prevalence was significantly higher in our gastric cancer cohort (4.4%) than in the general population (0.2%) (Table 2).

**Table 2:** Number of brain tumor cases in gastric cancer families with pathogenic *CDH1* variants (carriers and individuals at risk) compared to the estimated prevalence for brain tumors in the general population

	Gastric cancer families with pathogenic *CDH1* variant	General population (United States of America, 2010)	*p* value
Individuals with brain tumor	3 (4.4%)	688,096^a^ (0.2%)	0.0005***
Individuals without brain tumor	65	309,844,689	
Total	68	310,532,785^b^	

***p ≤ 0.001 (two-tailed Fisher’s exact test)

^a^According to Porter et al. (2010) [46]

^b^According to http://www.census.gov/popclock/ on December 31, 2010

As four of the five patients in our two glioma families carrying rare *CDH1* germline variants were affected by ODs, we investigated the frequency of *CDH1* variants in tumor DNA of a cohort of ODs, WHO grade II/III. Six of 99 (6%) ODs, WHO grade II/III were shown to harbor five different rare non-silent *CDH1* variants predicted to be deleterious, four missense and one nonsense variant, located in exons 12 (coding for EC5 of the E-cadherin ectodomain located in proximity to the cell membrane), 14 or 16 (coding for the intracellular domain), most of which were heterozygous and shown to be somatic (Suppl. Table 12 online resource). Interestingly, the c.1774G > A p.(A592T) variant, identified as a germline variant in glioma family 2, was also detected in the DNA of two ODs, WHO grade II/III. The MAF of each *CDH1* variant detected here in leukocyte or tumor DNA was at least four times higher, moreover, all but one variant was significantly more frequent in our glioma families or ODs, WHO grade II/III compared to gnomAD v2.1.1 controls (http://gnomad.broadinstitute.org/) (Suppl. Table 12 online resource). Characteristics of all OD patients and ODs and of their rare non-silent *CDH1* variants predicted to be deleterious are summarized in Suppl. Table 13 online resource.

A renal tumor NOS was diagnosed in patient I.1 of glioma family 1, and four of the six different *CDH1* variants detected in leukocyte DNA of glioma families or tumor DNA of ODs, WHO grade II/III were located in exons 14 to 16. Therefore, we performed targeted sequencing of *CDH1* exons 14 to 16 on tumor DNA of 65 renal cell carcinomas (RCC), the most common type of kidney cancer in adults. In one of 26 (4%) chromophobe RCCs, we detected the homozygous *CDH1*:c.2557T > C p.(S853P) variant predicted to be deleterious, absent in gnomAD controls, and located in exon 16 (coding for a part of the intracellular domain), while no rare *CDH1* variants were found in 21 clear cell RCCs or 18 papillary RCCs (Suppl. Table 14 online resource).

In summary, rare variants in the tumor suppressor gene *CDH1* were significantly more frequent in the germline of glioma families (13.3%) compared to controls (1.7%). Moreover, brain tumors were observed with a higher prevalence in gastric cancer families harboring pathogenic *CDH1* germline alterations (4.4%) than in the general population (0.2%). All brain tumors in *CDH1*-mutant glioma families were oligodendroglial tumors, and rare *CDH1* variants were detected in the tumor DNA of 6% of ODs, WHO grade II/III. Our data suggest an association of rare *CDH1* variants with the risk of brain tumors, particularly of gliomas, and with the tumorigenesis of ODs.

### *CDH1* is expressed in rat oligodendroglial cells and some human ODs, WHO grade II/III

**Fig. 2:**
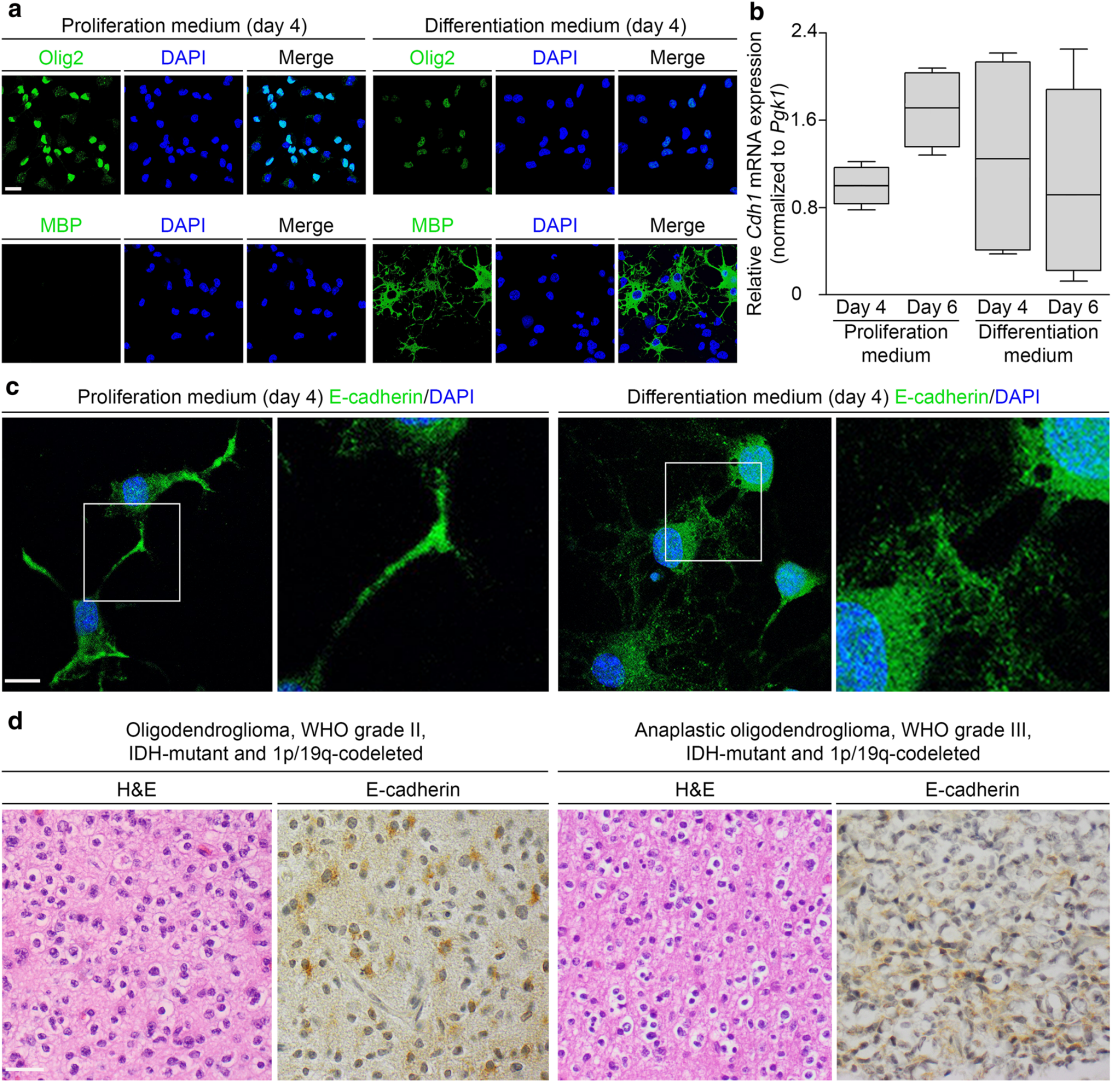
*CDH1* is expressed in rat primary oligodendroglial cultures and in some human ODs, WHO grade II/III. **a**–**c** *Cdh1* expression of primary oligodendroglial cultures isolated from neonatal Sprague–Dawley rats (P0–P3) and cultivated for four or six days in proliferation or differentiation medium was analyzed on the RNA and protein level. Prior to expression analysis, differentiation status of oligodendroglial cells was determined by immunostaining of oligodendrocyte transcription factor 2 (Olig2) and myelin basic protein (MBP). After incubation in proliferation medium, most cells were Olig2-positive while MBP was not detected, consistent with oligodendrocyte precursor cells; after incubation in differentiation medium, a substantial population of MBP-positive cells was observed, indicating differentiated oligodendrocytes; scale bar 15 µm (**a**). *Cdh1* mRNA was measured using SYBR green-based real-time RT-PCR and normalized to *Pgk1* mRNA. Appreciable levels of *Cdh1* mRNA expression were detected irrespective of medium and culture duration with the highest levels in oligodendroglial cultures after six days of proliferation medium. Expression data from four independent experiments performed in triplicate are presented as box plots (**b**). By immunofluorescence, E-cadherin expression in oligodendroglial cultures was localized to the cytoplasm and slightly enhanced at cellular extensions and regions of cell–cell contacts irrespective of culture medium used; scale bar 10 µm (**c**). By immunohistochemistry, E-cadherin was expressed in three of 19 (15.8%) human *CDH1* wildtype ODs, WHO grade II/III. Depicted are two E-cadherin-positive ODs, WHO grade II/III and consecutive hematoxylin–eosin (H&E) stained sections; scale bar 30 µm (**d**)

To investigate *Cdh1* expression at different stages of oligodendrocyte lineage development, we analyzed primary oligodendroglial cultures isolated from neonatal Sprague–Dawley rats after incubation with proliferation or differentiation medium for four or six days. In these cells, Olig2, expressed in oligodendrocyte precursor cells (OPCs), and MBP, expressed in mature oligodendrocytes, was visualized by immunofluorescence to characterize their differentiation status. After four days, the cells found in proliferation medium were Olig2-positive and MBP-negative, whereas in differentiation medium Olig2 staining was less intense and a substantial number of MBP-positive cells were observed, indicating that OPCs and immature oligodendrocytes were differentiating to mature oligodendrocytes (Fig. 2a). Using real-time RT-PCR analysis, *Cdh1* mRNA extracted from oligodendroglial cells was quantified showing appreciable *Cdh1* mRNA expression levels relative to *Pgk1* mRNA in all cultures, with the highest *Cdh1* levels after six days of proliferation medium in cultures containing OPCs and immature oligodendrocytes (Fig. 2b). Qualitative *Cdh1* mRNA analysis by direct sequencing of synthesized cDNA confirmed the presence of *Cdh1*-specific sequences in oligodendroglial culture-derived RNA samples (Suppl. Fig. 2 online resource). By immunostaining of oligodendroglial cells at day 4, E-cadherin was detected throughout the cytoplasm, slightly pronounced at cellular extensions and regions of cell–cell contacts irrespective of culture medium used (Fig. 2c). To assess E-cadherin expression in human ODs, immunohistochemistry was carried out on sections of *CDH1* wildtype ODs, WHO grade II/III. Three (one OD, WHO grade II and two anaplastic ODs, WHO grade III) of 19 ODs (15.8%) were immunopositive for E-cadherin (Fig. 2d). In summary, our findings that *CDH1* is expressed at the mRNA or protein level particularly in rat OPCs and immature oligodendrocytes, possible progenitor cells of OD, as well as in some human ODs, WHO grade II/III provide further evidence for a role of *CDH1* in OD risk and tumorigenesis.

### Identified *CDH1* germline variants affect cell membrane abundance of E-cadherin, cell adhesion and motility via altered structural dynamics of the E-cadherin ectodomain or β-catenin binding of the intracellular domain

**Fig. 3:**
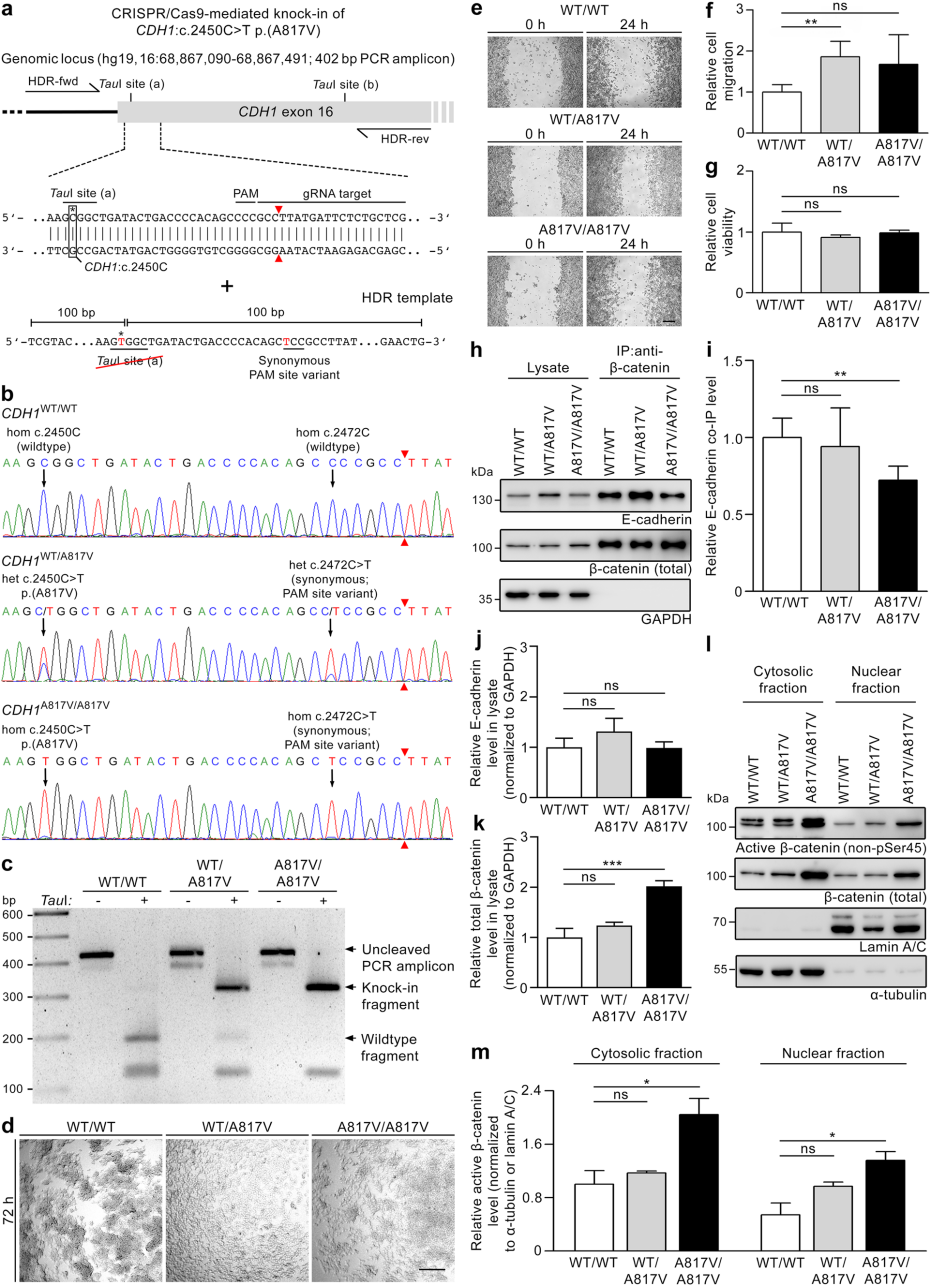
Generation and characterization of a HEK293T knock-in cell model harboring the *CDH1*:c.2450C > T p.(A817V) variant demonstrates its pathogenicity and affected pathomechanisms. **a** Schematic overview of the knock-in strategy using the CRISPR/Cas9 technology. The HDR template harboring the *CDH1* variant also contained a synonymous PAM site variant. **b** Electropherograms of the target region in *CDH1* exon 16 of the selected single cell clones showing two wildtype alleles (WT/WT), a heterozygous (WT/A817V) or homozygous (A817V/A817V) knock-in. The genotype of the A817V variant correlated with that of the synonymous PAM site variant. **c** The genotypes of the selected cell clones were confirmed by gel electrophoresis after *Tau*I restriction digest of a PCR amplicon from the target region in *CDH1* exon 16. **d** Using a slow aggregation assay, fewer cell–cell aggregates were observed in WT/A817V and A817V/A817V versus WT/WT cells after 72 h; representative images of one of three independent experiments performed in triplicate are shown; scale bar 200 µm. **e, f** In a wound healing assay analyzed 24 h after applying scratches, knock-in cells showed increased migration compared to WT/WT cells (**e**); relative cell migration (mean ± SD) was determined in four independent experiments performed in triplicate (**f**); scale bar 200 µm. **g** Using an MTS assay, no difference in cell viability was detected in knock-in compared to WT/WT cells after 44 h (mean ± SD of three independent experiments performed in triplicate). **h, i** By immunoprecipitation (IP) of β-catenin (**h**), a significant decrease of bound E-cadherin was detected in A817V/A817V compared to WT/WT cell lysates (mean ± SD of four independent experiments) (**i**) indicating impaired β-catenin binding of mutant E-cadherin. **j** By densitometric quantification of Western blot analyses (**h**), no differences in E-cadherin levels were observed in knock-in versus WT/WT cell lysates (mean ± SD of four independent experiments). **k** By densitometric quantification of Western blot analyses (**h**), total β-catenin levels were significantly increased in A817V/A817V versus WT/WT cell lysates (mean ± SD of four independent experiments). **l, m** Western blot analyses (**l**) and densitometric quantification (**m**) of the cytosolic and nuclear fractions showed a significant increase of active β-catenin levels in both fractions of A817V/A817V compared to WT/WT cells (mean ± SD of two independent experiments). *WT* wildtype; *p ≤ 0.05; **p ≤ 0.01; ***p ≤ 0.001; *ns* not significant (Student’s *t* test)

The potential pathogenicity of the rare *CDH1* variants identified in glioma families 1 and 2 was investigated using several in vitro and computational models. To analyze the consequences of the *CDH1*:c.2450C > T p.(A817V) variant, the CRISPR/Cas9 technology was utilized to generate genetically modified HEK293T cells (Fig. 3a). Genotypes of generated cell clones were verified by direct sequencing of the *CDH1* knock-in site and the gRNA target region confirming wildtype (WT/WT) status, heterozygous (WT/A817V) or homozygous (A817V/A817V) knock-in of selected clones (Fig. 3b). As part of the knock-in strategy (Fig. 3a), a synonymous variant was inserted into the PAM sequence of the HDR template to avoid Cas9 cleavage after successful repair and template incorporation, which was confirmed to be heterozygous in the WT/A817V clone and homozygous in the A817V/A817V clone (Fig. 3b). Due to the loss of one of the two *Tau*I sites in the HDR template containing the *CDH1*:c.2450C > T p.(A817V) variant, cell clone genotypes could also be verified by gel electrophoresis after a restriction digest (Fig. 3a, c). In the three selected cell clones, no additional off-target variation was detected at the predicted coding gRNA target sites by direct sequencing (Suppl. Table 15 online resource).

Using the genetically modified HEK293T cell model to analyze the effect of the *CDH1*:c.2450C > T p.(A817V) variant, we could show reduced cell aggregation in heterozygous and homozygous knock-in cells compared to the wildtype clone by slow aggregation assay (Fig. 3d). In a wound healing assay, HEK293T cell migration was enhanced in the heterozygous and homozygous knock-in clones compared to the wildtype clone (Fig. 3e–f). A cell proliferation-dependent effect was excluded because the three HEK293T cell clones had comparable relative cell viability values in an MTS assay (Fig. 3g). As the *CDH1*:c.2450C > T p.(A817V) variant is located in the β-catenin-associated intracellular region of E-cadherin, we explored a possible effect of the variant on the interaction of E-cadherin with β-catenin. By immunoprecipitation, mutant E-cadherin binding to β-catenin in A817V/A817V HEK293T cell lysates was significantly decreased compared to that of wildtype E-cadherin in WT/WT cell lysates (Fig. 3h–i). When comparing E-cadherin and total β-catenin levels in cell lysates (Fig. 3h), E-cadherin levels were comparable in WT/WT, heterozygous and homozygous knock-in cells (Fig. 3j), while a significant increase of total β-catenin levels in A817V/A817V versus WT/WT cell lysates was detected (Fig. 3k). Levels of active β-catenin were also enhanced in A817V/A817V compared to WT/WT cells, as shown for their cytosolic and nuclear fraction (Fig. 3l–m), while the ratios of active to total β-catenin levels in these cell fractions did not differ in heterozygous and homozygous knock-in compared to WT/WT clones (Suppl. Fig. 3 online resource). These data suggest that the *CDH1*:c.2450C > T p.(A817V) variant compromises the effect of E-cadherin on cell aggregation and migration by reducing the interaction with β-catenin and increasing β-catenin levels in the cytosol and the nucleus.

**Fig. 4:**
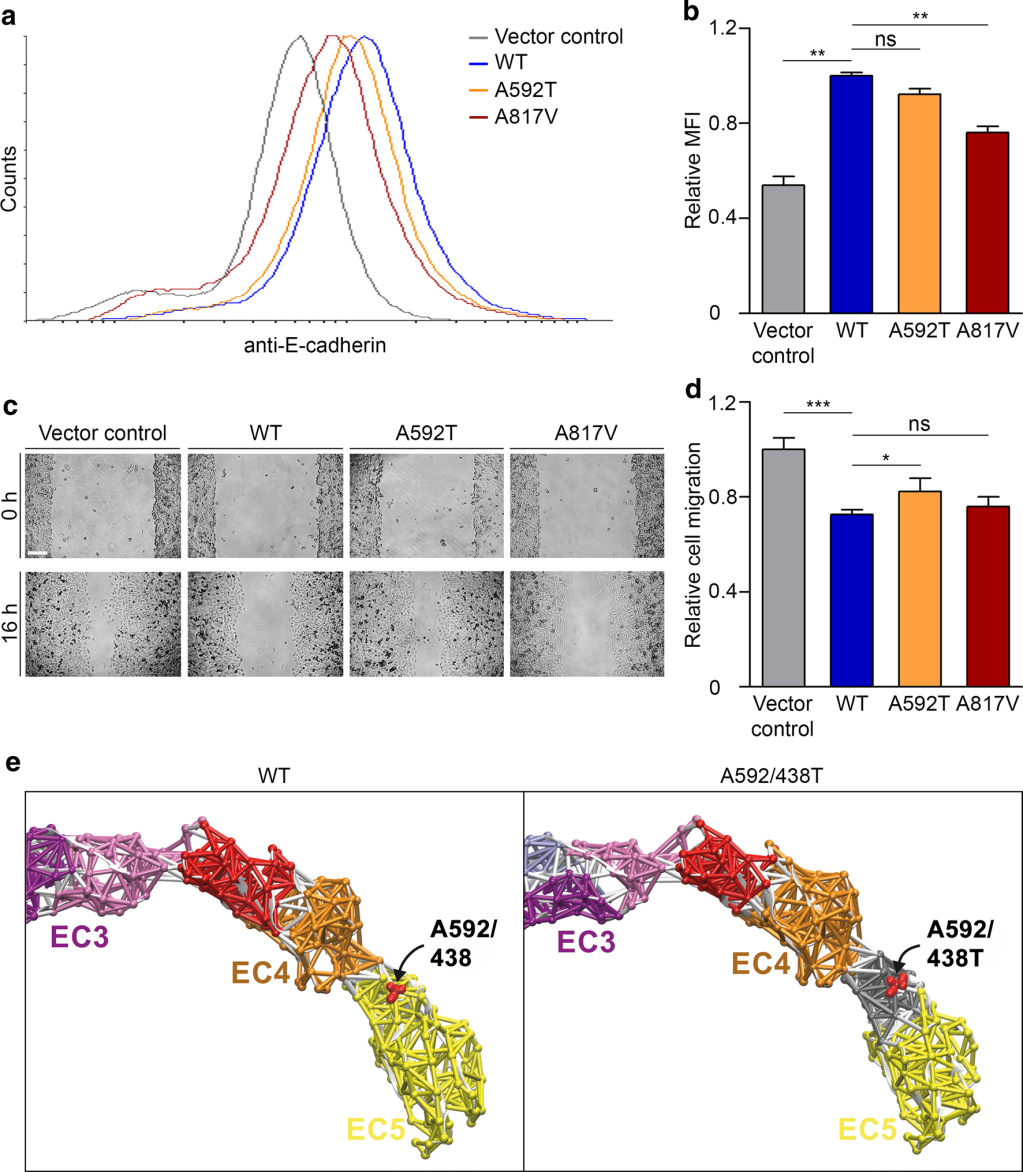
A cellular model using CHO cells stably expressing E-cadherin harboring the A592T or A817V variants and a computer simulation demonstrate variant pathogenicity and affected pathomechanisms. **a, b** Representative histograms (smoothed) obtained by flow cytometry of anti-E-cadherin stained CHO cells transfected with empty vector (vector control, *grey line*) or stably expressing E-cadherin WT (*blue line*), E-cadherin A592T (*orange line*), or E-cadherin A817V (*red line*) (**a**). Quantification of flow cytometry experiments revealed a significant decrease in relative median fluorescence intensity (MFI) in cells expressing E-cadherin A817V compared to E-cadherin WT, indicating lower cell membrane abundance of E-cadherin A817V (mean ± SD of three independent experiments performed in duplicate) (**b**). **c, d** Cell migration was analyzed in stably transfected CHO cells 16 h after applying scratches; scale bar 150 µm (**c**). Quantification of the wound healing assay showed a significant increase in migration of cells expressing E-cadherin A592T compared to E-cadherin WT (mean ± SD of three independent experiments performed in triplicate) (**d**). **e** Dynamical network analysis of the 100 ns MD simulations of murine E-cadherin WT and A592/438T ectodomains (also see Suppl. Fig. 6 online resource). Regions of correlated motion are individually colored according to the clustered network communities as shown here for extracellular domains EC3 to EC5. EC5 of E-cadherin WT with a conserved alanine residue (shown as *red spheres*) at position 592 (human pre-protein)/438 (mature protein), located at the interface with EC4 (*red* and *orange* communities), is represented by one network community (*yellow*). The threonine variant (shown as *red spheres*) at position 592 (human pre-protein)/438 (mature protein) in E-cadherin A592/438T led to changes in the network communities, with a new community (*grey*) identified around the variant site, indicating uncoupling of spatiotemporal motions. *p ≤ 0.05; **p ≤ 0.01; ***p ≤ 0.001; ns, not significant (Student’s *t* test)

To analyze the consequence of the *CDH1*:c.1774G > A p.(A592T) variant in addition to that of the *CDH1*:c.2450C > T p.(A817V) variant, we generated a second cellular model using CHO cells that do not endogenously express E-cadherin. CHO cells were stably transfected with empty vector (vector control) or constructs expressing wildtype or mutant (A592T or A817V) E-cadherin, resulting in comparable total E-cadherin expression levels (Suppl. Fig. 4 online resource). The generated cell lines were examined with regard to E-cadherin cell membrane expression, cell migration and aggregation. By flow cytometry of fixed anti-E-cadherin-stained CHO cells, the median fluorescence intensity was significantly decrease in cells expressing E-cadherin A817V compared to wildtype E-cadherin, indicating a lower abundance of E-cadherin A817V at the cell membrane (Fig. 4a,b). In a wound healing assay, cell migration was significantly enhanced in E-cadherin A592T versus wildtype E-cadherin expressing CHO cells, while E-cadherin A817V expressing cells only showed a slight increase (Fig. 4c,d). Using a slow aggregation assay and comparable to our findings in HEK293T knock-in cells (Fig. 3d), cell aggregation was diminished in CHO cells expressing E-cadherin A817V compared to wildtype E-cadherin, while E-cadherin A592T did not affect aggregation of CHO cells (Suppl. Fig. 5 online resource). These data corroborate and extend our findings in the HEK293T knock-in cell model regarding pathogenicity of the *CDH1*:c.2450C > T p.(A817V) variant, and provide experimental evidence that the *CDH1*:c.1774G > A p.(A592T) variant is a hypomorph with respect to cell migration.

To gain further insights into the impact of the *CDH1*:c.1774G > A p.(A592T) variant and to explore the pathomechanism involved, we performed all-atom MD simulations in explicit water starting from the crystal structure of the murine E-cadherin ectodomain (82% sequence identity to the human E-cadherin ectodomain). The human A592T variant was inserted at the corresponding conserved position 438 (A592/438T), located between EC4 and EC5 of the ectodomain (Fig. 4e, Suppl. Fig. 6 online resource), into the crystal structure of the mature murine protein using the Schrödinger Suite. Simulations of the wildtype E-cadherin ectodomain served as reference. Analysis of the 100 ns MD simulations indicated a markedly increased flexibility of E-cadherin A592/438T as compared to wildtype, leading to large spatiotemporal deviations of the ectodomain, particularly of EC1, EC4, and EC5 (Suppl. Fig. 6 online resource). During our simulations, the ectodomain of E-cadherin A592/438T featured a much stronger structural bending with higher positional fluctuations. To better understand this effect, we constructed dynamical network models [55] according to the degree of coupled motions of protein residues during the MD simulations. Clustering using the Girvan–Newman algorithm [17] revealed individual network communities representing structural subregions showing correlated motions in the ectodomain. The obtained network communities correlated well with the architecture of the ectodomain with one or two communities per extracellular domain (Suppl. Fig. 6 online resource). Network analysis of E-cadherin A592/438T simulations indicated that the variant primarily altered the network communities of domains EC1 and EC5 (Suppl. Fig. 6 online resource, Fig. 4e). The region around the variant site clustered as an individual network community (Fig. 4e) suggesting an uncoupling of that subregion from the remaining community of the EC5 domain, which might explain the increased spatiotemporal flexibility of EC4 and EC5. Our findings suggest an impact of the A592T variant on the structural dynamics of the E-cadherin ectodomain, whereby it may destabilize protein conformations required for adhesion to other ectodomains from adjacent cells.

Using two cellular models and computer simulations, we have provided evidence that the identified *CDH1* germline variants c.1774G > A p.(A592T) and c.2450C > T p.(A817V) act as hypomorphs affecting cell membrane abundance of E-cadherin, cell migration or cell–cell adhesion, possibly via altered structural dynamics of the ectodomain or β-catenin binding of the intracellular domain and cellular β-catenin levels.

### Targeting the effects of the identified *CDH1* germline variants by treatment with the MNK1 inhibitor CGP 57380

**Fig. 5:**
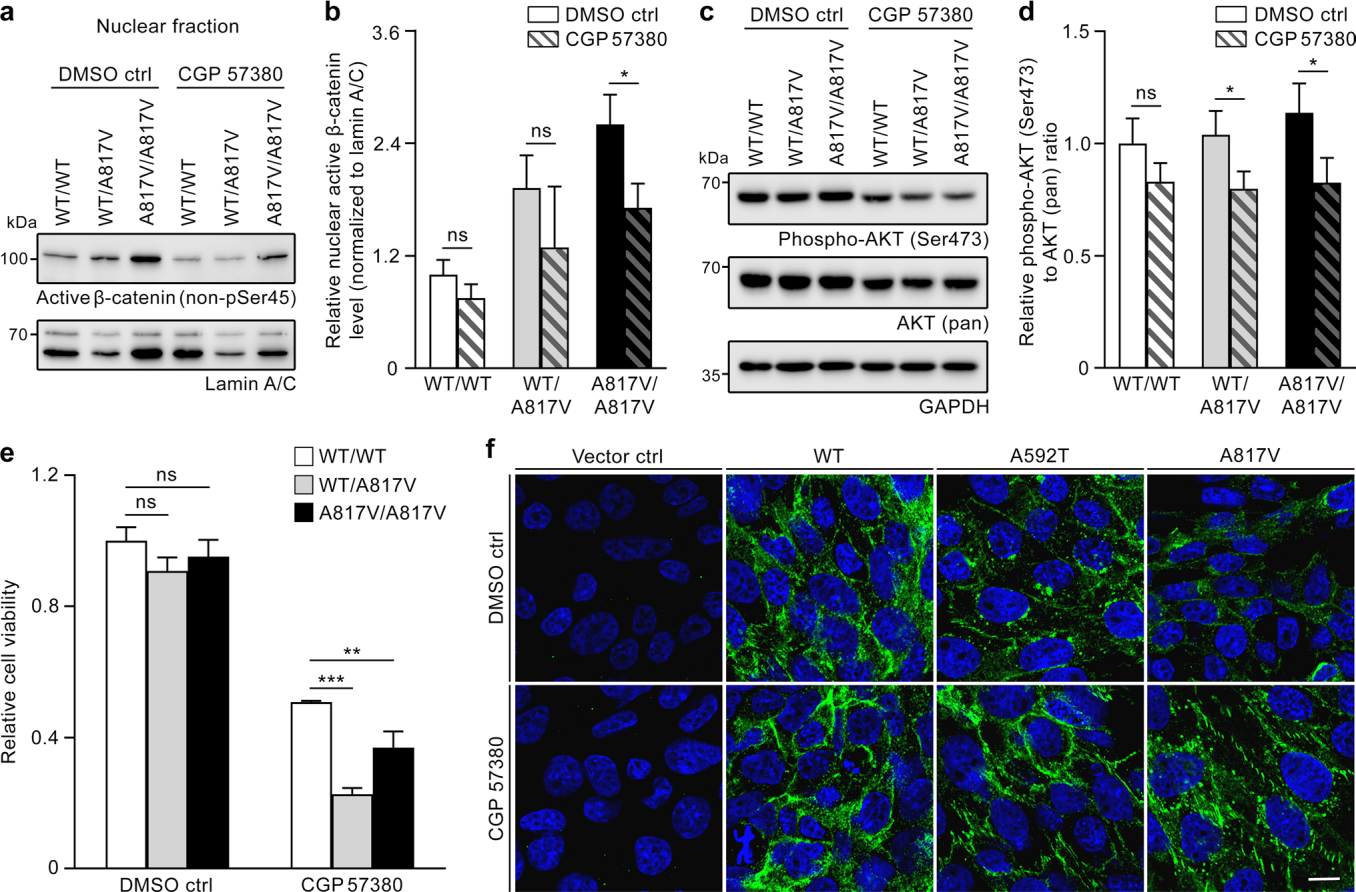
Treatment with the MNK1 inhibitor CGP 57380 reverses effects of the identified deactivating *CDH1* variants in two cellular models. **a** Western blot analysis of active β-catenin (detected using a non-phospho-Ser45-specific antibody) in the nuclear fraction of heterozygous (WT/A817V) and homozygous (A817V/A817V) E-cadherin A817V knock-in and E-cadherin wildtype (WT/WT) HEK293T clones after 24 h incubation with CGP 57380 (75 µM) or DMSO control. **b** Densitometric evaluation of protein bands from (**a**) revealed a CGP 57380-dependent significant reduction of nuclear active β-catenin normalized to lamin A/C for the A817V/A817V clone, but not for the WT/A817V or WT/WT clones (mean ± SD of three independent experiments). **c** Western blot analysis of phospho-AKT (Ser473) and AKT (pan) in lysates of A817V knock-in and E-cadherin wildtype HEK293T clones after 24 h treatment with CGP 57380 (75 µM) or DMSO control.** d** Densitometric evaluation of the protein bands from (**c**) revealed a CGP 57380-dependent significant reduction of the ratio of phospho-AKT (Ser473) to AKT (pan) levels in WT/A817V and A817V/A817V cells, but not in WT/WT cells (mean ± SD of three independent experiments). **e** By MTS assay, cell viability after treatment with CGP 57380 (200 µM) for 72 h was significantly lower in WT/A817V and A817V/A817V cells than in WT/WT cells (mean ± SD of three independent experiments). **f** Detection of E-cadherin in CHO cells stably expressing E-cadherin WT, A592T or A817V after incubation with DMSO control or CGP 57380 by immunofluorescence. The reduced cell membrane localization of mutant E-cadherin observed after incubation with DMSO control could be reversed by treatment with CGP 57380 (75 µM) for 24 h; representative images of each cell line of one of three independent experiments are shown; scale bar 10 µm. *Ctrl* control; *p ≤ 0.05; **p ≤ 0.01; ***p ≤ 0.001; *ns* not significant (Student’s *t* test)

We used the generated cell models to test a therapeutic approach particularly aimed at individuals carrying the *CDH1* missense variants identified in glioma families 1 and 2. HEK293T A817V knock-in cells exhibited higher nuclear levels of active β-catenin compared to WT/WT cells (Fig. 3l,m), and treatment with the MNK1 inhibitor CGP 57380 downregulated β-catenin levels in the nucleus of nasopharyngeal carcinoma cell lines [62]. Therefore, we investigated the effect of CGP 57380 on the nuclear translocation of active β-catenin in our HEK293T knock-in cell model. In A817V/A817V cells, treatment with CGP 57380 significantly reduced the nuclear levels of active β-catenin as compared to DMSO control, while nuclear levels in WT/WT and WT/A817V cells were not significantly affected by the inhibitor (Fig. 5a,b). In nasopharyngeal carcinoma cells, inhibition of β-catenin nuclear translocation by CGP 57380 was dependent on AKT activation [62]. Therefore, we analyzed phospho-Ser473 AKT (active form) in our HEK293T knock-in cell model. In response to CGP 57380 versus DMSO control, we detected a significant reduction of AKT phosphorylation as determined by the ratio of phosphorylated AKT to AKT (pan) in heterozygous and homozygous A817V knock-in clones, but not in the WT/WT clone (Fig. 5c,d). Possibly as a consequence of the diminished levels of nuclear β-catenin and phosphorylated AKT caused by CGP 57380 treatment, the MNK1 inhibitor significantly reduced the viability of heterozygous and homozygous A817V knock-in cells as compared to WT/WT cells (Fig. 5e). Moreover, CGP 57380 treatment of stably transfected CHO cells resulted in increased cell membrane localization of E-cadherin, as shown by immunofluorescence. Thereby, the reduced membrane-associated staining observed in DMSO-treated cells stably expressing E-cadherin A592T or A817V was enhanced after treatment with CGP 57380 (Fig. 5f). Our data suggest that molecular alterations caused by the identified *CDH1* variants, such as reduced E-cadherin cell membrane abundance and elevated nuclear active β-catenin, can be reverted by treatment with the MNK1 inhibitor CGP 57380.

## Discussion

To date, heterozygous *CDH1* germline mutations have been described to be causative of specific types of hereditary cancer, mainly diffuse gastric cancer [20] and invasive lobular breast cancer [63], and on the other hand of congenital malformations involving the face, i.e. blepharocheilodontic syndrome and cleft lip/palate [14]. Here, we provide evidence that in addition to the phenotype spectrum previously described, rare *CDH1* germline aberrations may predispose to neuroepithelial and epithelial brain tumors. Thus, rare *CDH1* germline variants predicted to be deleterious co-segregated with the tumor phenotype, mostly with ODs, WHO grade II/III, in two of 15 (13.3%) glioma families, and their frequency was significantly lower in controls (1.7%). In a cohort of 28 gastric cancer families with pathogenic *CDH1* germline variants, brain tumors were observed in three of 68 (4.4%) individuals, a significantly higher prevalence than in the general population (0.2%). In line with our findings, brain tumors have previously been reported in gastric cancer families with causative germline alterations in *CDH1* [28] and other (suspected) tumor suppressor genes, e.g. *ATM*, *CTNNA1*, and *SDHB* [21]. Similarly, most *CDH1* variants identified here in glioma patients or glioma tissues were previously detected in the germline of patients with cancer of the gastrointestinal tract, such as gastric or colorectal cancer (references listed in Suppl. Table 13 online resource). Taken together, these data support the notion that some genetic alterations predisposing to tumors of the gastrointestinal tract may also increase the risk of brain tumors. Further evidence for this view comes from cancer predisposition syndromes with a tumor spectrum including tumors of the gastrointestinal tract and the nervous system, e.g. Cowden, Li-Fraumeni, Lynch syndromes, familial adenomatous polyposis [30], and familial intestinal gastric cancer [11], suggesting that pathogenic germline variants in the underlying genes, e.g. *PTEN*, *TP53*, *MSH2*, *MSH6*, *MLH1*, *PMS2*, *APC*, cause tumors in both organ systems. Although based on a limited number of families requiring confirmation in larger cohorts, our data provide evidence that this list may be extended to the hereditary diffuse gastric cancer (HDGC) syndrome and the *CDH1* gene.

More specifically, our data imply that ODs are the brain tumors most likely to occur in the presence of a rare *CDH1* germline variant, because this tumor type was observed in four of five tumor patients carrying rare *CDH1* germline variants of two glioma families here. This finding is of particular significance since ODs, WHO grade II/III are quite rare, accounting for only 1.3% of all primary CNS tumors [43]. To date, little is known about germline alterations predisposing to ODs. In a large case–control study, a low-frequency variant at 8q24.21 was strongly associated with the risk of ODs, but also of IDH-mutant astrocytomas [25]. By analyzing glioma families, rare highly penetrant germline variants associated mainly with a predisposition for ODs were described in the *POT1* gene [2], although not detected in this study. The role of *CDH1* in OD risk identified here is corroborated by our finding that rare non-silent *CDH1* variants predicted to be deleterious were also observed in tumor DNA of 6% of ODs, WHO grade II/III.

*CDH1* expression in glial cells and gliomas has not been comprehensively studied. In rat Schwann cells co-cultured with dorsal root ganglion neurons, E-cadherin is expressed under nonmyelinating and myelinating conditions, and promotes Schwann cell myelin formation [3], indicating that E-cadherin plays a role in glial cells of the peripheral nervous system. Here, by analyzing oligodendroglial cultures prepared from the rat CNS, we detected E-cadherin expression on the transcript and protein level particularly in OPCs and differentiating oligodendrocytes. Our findings are consistent with transcriptome and proteome studies of brain cell type-specific expression patterns in mouse and humans using RNA sequencing and high-resolution mass spectrometry, where *CDH1* mRNA or E-cadherin were found predominantly in oligodendrocytes [56, 64]. Given that OPC populations that do not differentiate to mature myelinating oligodendrocytes are maintained in the adult CNS, comprising around 10% of its cellular content, and retain their proliferative and migratory potential, OPCs possess the properties and the number required for a glioma cell of origin [31]. Introducing *Tp53*/*Nf1* mutations into OPCs in a mouse model consistently led to gliomagenesis, providing experimental data that gliomas may arise by malignant transformation of OPCs [35]. Besides showing *CDH1* expression in OPCs, i.e. cells implicated in gliomagenesis, we and others [12, 41] detected immunoreactivity for E-cadherin in ODs, providing evidence that *CDH1* variants, such as those reported here, may impact these cells or tissues. Furthermore, in low-grade gliomas, including ODs, *CDH1* is frequently altered by an alternative mechanism, i.e. hypermethylation of its promoter, resulting in down-regulated E-cadherin expression and worse outcome of patients [12]. *CDH1* promoter hypermethylation may explain why we only found E-cadherin to be expressed in a subset of ODs, WHO grade II/III, and further supports a role of *CDH1* in gliomagenesis.

To assess the consequences of the two rare *CDH1* germline variants, c.1774G > A p.(A592T) and c.2450C > T p.(A817V), identified in glioma families 1 and 2, we performed functional analyses using two cellular models, i.e. CRISPR/Cas9-mediated HEK293T knock-in and stably transfected CHO cells. Both variants showed an effect on cell migration, and the A817V variant additionally had an impact on cell membrane expression of E-cadherin, cell aggregation, and interaction with β-catenin. In contrast, a previous report failed to show a functional consequence of the A592T variant [29]. Although this variant has been reported in patients with familial gastric cancer [15, 29, 52], invasive lobular breast cancer [60], hereditary prostate cancer [27], and colorectal cancer [52], it has also been identified in cancer-unaffected individuals [15, 29, 52], is not extremely infrequent (MAF of just under 0.3% according to gnomAD controls), and was classified as benign in the human variation and phenotype database ClinVar by many submitters. However, co-segregation of the A592T variant with the tumor phenotype in glioma/cancer families was shown here and previously [27, 52], and we detected this variant in two of 75 ODs, WHO grade II/III tested, more than four times more frequently than in controls. In contrast to the limited effect of the alanine to threonine amino acid exchange at position 592/438 predicted in the molecular model of the murine E-cadherin ectodomain by others [15], we could show by MD simulations using the same crystal structure that the E-cadherin A592/438T ectodomain featured a much stronger structural bending with higher positional fluctuations compared to the wildtype ectodomain. Such an increased flexibility might negatively affect interactions of E-cadherin ectodomains in *trans*, and, therefore, cell–cell adhesion by disturbing the shape complementarity and proper alignment of the interaction interfaces of the ectodomains from different cells. Moreover, binary interactions in *cis*, e.g. with glioma-associated proteins, such as the EGF receptor (EGFR) [47], may also be affected by increased fluctuation. Thus, the E-cadherin A592T variant may enhance ligand-mediated activation of EGFR, known to be inhibited by wildtype E-cadherin (E-cadherin-EGFR cross-talk) [33, 47]. This could represent an alternative mechanism to the activation of EGFR signaling further downstream by inactivation of the *CIC* gene, encoding a transcriptional repressor of EGFR-dependent genes [57], which frequently occurs in ODs by *CIC* mutation [7]. The A817V variant, previously described in patients with familial colorectal cancer before 60 years of age [50], has not been functionally characterized. Here, we propose that the effect of E-cadherin harboring the A817V variant is mediated by reduced β-catenin binding, as previously shown for the V832M variant [44], and increased β-catenin levels available for WNT/β-catenin signaling. Our findings are in line with previous data linking the up-regulation of the WNT/β-catenin signaling pathway to the development of certain brain tumors, e.g. medulloblastomas frequently harboring mutations in the *CTNNB1* gene encoding β-catenin [58] and glioblastomas [59]. In a recent pan-cancer analysis of oncogenic pathways, WNT signaling was shown to be altered in around two-thirds of IDH-mutant, 1p/19q-codeleted low-grade gliomas, i.e. ODs according to the WHO classification of 2016 [37], in that they exhibited inactivation of the *TCF7* gene [53]. *TCF7* inactivation and *CDH1* variants affecting β-catenin binding, detected in ODs previously and here, are different alterations that may lead to the same downstream effect, i.e. enhancement of WNT/β-catenin signaling [1]. As activation of EGFR signaling was also shown to promote nuclear translocation of β-catenin [32], the E-cadherin A592T and A817V germline variants may both impact the WNT/β-catenin pathway. This finding is of particular relevance because WNT/β-catenin signaling is known to inhibit OPC differentiation into mature oligodendrocytes [4]. Therefore, OPCs, which were shown to express E-cadherin here, existing in the adult brain [13] may remain undifferentiated instead of maturing if they harbor E-cadherin A592T and A817V variants, predisposing to malignant transformation. Collectively, these data link the *CDH1* germline variants detected here to OD predisposition via activation of the WNT/β-catenin pathway.

Consequently, WNT/β-catenin signaling was pharmacologically targeted in the two generated cellular models expressing the E-cadherin A592T and A817V germline variants by MNK1 inhibition with CGP 57380. The MNK-eukaryotic translation initiation factor 4E axis has been reported to activate β-catenin signaling in blast crisis chronic myeloid leukemia [34]. Accordingly, the MNK1 inhibitor CGP 57380 was effective in preventing nuclear translocation of β-catenin in models of chronic myeloid leukemia and nasopharyngeal carcinoma in vitro and in vivo [34, 62]. Moreover, CGP 57380 in combination with mTORC1 inhibition or temozolomide treatment has shown preclinical efficacy in glioma cells, an orthotopic glioblastoma mouse model or glioblastoma-derived spheres [18, 19]. In our cellular models expressing the E-cadherin variants identified in the germline of glioma families, CGP 57380 diminished nuclear β-catenin and the viability of these cells, and enhanced E-cadherin cell membrane abundance. Therefore, sensitizing gliomas with MNK1 inhibitors prior to e.g. temozolomide treatment may be particularly effective in glioma patients carrying *CDH1* variants affecting WNT/β-catenin signaling.

In the glioma families carrying deactivating *CDH1* germline variants here, we observed no cases of gastric or breast cancer. Instead, the non-glial tumors consisted of a renal tumor and a serous ovarian carcinoma. Although the tumor spectrum seen does not correspond to that typically detected in the HDGC syndrome, ovarian carcinoma has been described in patients carrying the *CDH1*:c.1018A > G p.(T340A) variant [14]. Considering the tumor types observed in our glioma families, recommending prophylactic total gastrectomy as an option, as is done in carriers of unequivocally pathogenic *CDH1* variants [9], does not seem appropriate. However, in recent studies of individuals with pathogenic *CDH1* variants, the family history was not a reliable determinant of the diffuse gastric cancer risk [24, 61], making a sound recommendation difficult.

In summary, our results link heterozygous *CDH1* variants that compromise the tumor suppressor function of E-cadherin and may affect WNT/β-catenin signaling to brain tumor, primarily to OD, risk and tumorigenesis, providing evidence that the phenotype spectrum of rare *CDH1* germline variants may extend to brain tumors of neuroepithelial and epithelial origin. Furthermore, we propose that pharmacologically targeting WNT/β-catenin signaling by MNK1 inhibitors may have prophylactic or therapeutic potential specifically in (oligodendro)glioma patients harboring *CDH1* variants affecting this pathway.

## Supplementary Information

Below is the link to the electronic supplementary material.

Supplementary file1 (PDF 1694 KB)
