# Supplementary material for: Rare germline variants in the E-cadherin gene CDH1 are associated with the risk of brain tumors of neuroepithelial and epithelial origin
Source: Acta Neuropathol. 2021 Apr 30;142(1):191–210. doi: 10.1007/s00401-021-02307-1 (PMC8217027; doi:10.1007/s00401-021-02307-1)
Supplement: Supplementary file 1 — Supplementary file1 (PDF 1694 KB) [file 401_2021_2307_MOESM1_ESM.pdf]

ACTA NEUROPATHOLOGICA

SUPPLEMENTARY MATERIAL

Rare germline variants in the E-cadherin gene *CDH1* are associated with the risk of brain tumors of neuroepithelial and epithelial origin

Alisa Förster[1*], Frank Brand[1*], Rouzbeh Banan[2,3], Robert Hüneburg[4,5], Christine A. M. Weber[1], Wiebke Ewert[6], Jessica Kronenberg[7,8,9], Christopher Previti[10,11], Natalie Elyan[1], Ulrike Beyer[1], Helge Martens[1], Bujung Hong[12], Jan H. Bräsen[13], Andreas Erbersdobler[14], Joachim K. Krauss[12], Martin Stangel[7,8], Amir Samii[15], Stephan Wolf[10], Matthias Preller[6,16], Stefan Aretz[4,17], Bettina Wiese[12,18], Christian Hartmann[2], Ruthild G. Weber[1]

[1]Department of Human Genetics, Hannover Medical School, Hannover, Germany
[2]Department of Neuropathology, Institute of Pathology, Hannover Medical School, Hannover, Germany
[3]Department of Neuropathology, Institute of Pathology, University Hospital Heidelberg, Heidelberg, Germany
[4]National Center for Hereditary Tumor Syndromes, University Hospital Bonn, Bonn, Germany
[5]Department of Internal Medicine I, University Hospital Bonn, Bonn, Germany
[6]Institute for Biophysical Chemistry, Hannover Medical School, Hannover, Germany
[7]Clinical Neuroimmunology and Neurochemistry, Department of Neurology, Hannover Medical School, Hannover, Germany
[8]Center for Systems Neuroscience, University of Veterinary Medicine Hannover, Hannover, Germany
[9]German Aerospace Centre (DLR), Institute of Aerospace Medicine, Radiation Biology Department, Köln, Germany
[10]Genomics and Proteomics Core Facility, High Throughput Sequencing Unit W190; German Cancer Research Center (DKFZ), Heidelberg, Germany
[11]Omics IT and Data Management Core Facility W610, German Cancer Research Center (DKFZ), Heidelberg, Germany
[12]Department of Neurosurgery, Hannover Medical School, Hannover, Germany
[13]Nephropathology, Institute of Pathology, Hannover Medical School, Hannover, Germany
[14]Institute of Pathology, University of Rostock, Rostock, Germany
[15]Department of Neurosurgery, International Neuroscience Institute, Hannover, Germany
[16]Department of Natural Sciences, University of Applied Sciences Bonn-Rhein-Sieg, Germany
[17]Institute of Human Genetics, Medical Faculty, University of Bonn, Bonn, Germany
[18]Department of Neurology, Henriettenstift, Diakovere Krankenhaus gGmbH, Hannover, Germany

*Alisa Förster and Frank Brand contributed equally as first authors to this work.

Correspondence to: Ruthild G. Weber, M.D., Department of Human Genetics OE 6300, Hannover Medical School, Carl-Neuberg-Str. 1, 30625 Hannover, Germany, Phone +49 511 532 7751, Fax +49 511 532 18520, Email: Weber.Ruthild@mh-hannover.de

Short running title: *CDH1* germline variants in brain tumor risk

SUPPLEMENTARY MATERIALS AND METHODS

Isolation of rat oligodendroglial cells

To prepare primary oligodendroglial cultures from neonatal Sprague-Dawley rats (P0-P3), meninges, choroid plexus, cerebellum and brain stem were removed, and brains were dissociated mechanically and digested enzymatically with 0.1% trypsin (Biochrom, Berlin, Germany) and 0.25% DNase (Roche, Basel, Switzerland). The obtained single cell suspensions were seeded into culture flasks pre-coated with poly-L-lysine (Sigma-Aldrich, St. Louis, MO, USA). Cultures were kept in Dulbecco's Modified Eagle Medium (DMEM; Thermo Fisher Scientific, Waltham, MA, USA) supplemented with 1% penicillin/streptomycin (Sigma-Aldrich) at 37°C and 5% CO_2 until use. On day 7, microglial cells were harvested after shaking the culture flasks at 180 rpm and 37°C for 40 min on an orbital shaker. After allowing the remaining cells in the culture flasks to rest for at least 2 h, oligodendroglial cells were isolated by orbitally shaking the culture flasks at 160 rpm and 37°C for 16-20 h. Supernatants were collected, centrifuged and, to reduce contamination with astrocytes and microglia, cells were kept in uncoated culture flasks for 20-30 min at 37°C. Medium containing free-floating oligodendroglial cells was collected, centrifuged, and cells were seeded on glass coverslips coated with poly-L-lysine and kept in medium until day 4 and 6, whereby 80,000 cells were plated in proliferation medium: KnockOut™ DMEM/F-12 supplemented with GlutaMAX™, StemPro supplement, EGF, human FGF, PDGF-AA (all from Thermo Fisher Scientific), and 100,000 cells were plated in differentiation medium: Neurobasal® medium supplemented with GlutaMAX™, B-27 supplement (all from Thermo Fisher Scientific), and 30 ng/ml T3 (Sigma-Aldrich).

Antibodies used for immunofluorescence microscopy

The following primary antibodies were used for immunostaining of oligodendroglial (precursor) cells: mouse anti-E-cadherin (#610181, Becton, Dickinson and Company, Franklin Lakes, NJ, USA; dilution 1:50), rabbit anti-Olig2 (#AB9610, Sigma-Aldrich; dilution 1:500), and mouse anti-MBP (#SMI99, BioLegend, San Diego, CA, USA; dilution 1:500).

CRISPR/Cas9-mediated knock-in of *CDH1*:c.2450C>T p.(A817V)

To generate a knock-in of *CDH1*:c.2450C>T p.(A817V) in HEK293T cells, the synthesized guide RNA (gRNA) was inserted by T4 DNA ligase into a *Bpi*I-digested pSpCas9(BB)-2A-GFP plasmid (#48138, Addgene, Watertown, MA, USA) containing a gRNA scaffold and expression cassettes for Cas9 and GFP. By transient transfection, the Cas9/gRNA construct and the homology-directed repair template were introduced into HEK293T cells. After 24 hours, GFP-positive cells were isolated using a MoFlo XDP cell sorter (Beckman-Coulter, Brea, MA, USA), and their genomic DNA was extracted using the innuPREP DNA Mini Kit (Analytik Jena, Jena, Germany). To identify the knock-in genotype of selected cell clones, PCR products of *CDH1* exon 16 were analyzed by (i) direct sequencing and (ii) agarose gel electrophoresis after 16 h treatment with *Tau*I restriction enzyme to verify the loss of one of two *Tau*I restriction sites due to the introduced *CDH1*:c.2450C>T variant.

Primary antibodies used for Western blot analysis

The following primary antibodies were used for immunodetection: anti-AKT (pan) (#4691, C67E7, Cell Signaling Technology (CST), Danvers, MA, USA; dilution 1:1,000), anti-phospho-AKT (Ser473) (#4060, D9E, CST; dilution 1:1,000), anti-E-cadherin (#3195, 24E10, CST; dilution 1:1,000), anti-β-catenin (#2698, L87A12, CST; dilution 1:1,000), anti-non-phospho-Ser45-(active)-β-catenin (#19807, D2U8Y, CST; dilution 1:1,000), anti-GAPDH (MAB374, 6C5, Merck; dilution 1:3,000), anti-GFP (sc-9996, Santa Cruz Biotechnology, Dallas, TX, USA; dilution 1:1,000), anti-lamin A/C (#4777, 4C11, CST; dilution 1:1,000) or anti-α-tubulin (#2125, 11H10, CST; dilution 1:1,000).

Molecular dynamics simulations

For molecular dynamics simulations, the crystallized sequence of the ectodomain of wildtype and mutant E-cadherin were fully solvated with the TIP3P explicit water model (Jorgensen et al. 1983), and the systems were neutralized by adding Na^+ counter ions. A minimum distance of 9 Å between the protein and water box edges was used with periodic boundary conditions.

Langevin dynamics and the Langevin piston method maintained a constant temperature of 310 K and a constant pressure of 1 atm. A cutoff of 12 Å was used for van-der-Waals and short-range electrostatics, and the particle-mesh Ewald method (Darden et al. 1993) was used for long-range electrostatic interactions. The solvated systems were initially energy-minimized and equilibrated for at least 5 ns, prior to 100 ns production runs with an integration time step of 2 fs. All MD simulations were carried out on the supercomputer of the North-German Supercomputing Alliance (HLRN). Dynamical network analysis was carried out according to Sethi et al. (2009). Simulation trajectory analysis was performed using VMD 1.9 (Humphrey et al. 1996).

SUPPLEMENTARY FIGURES

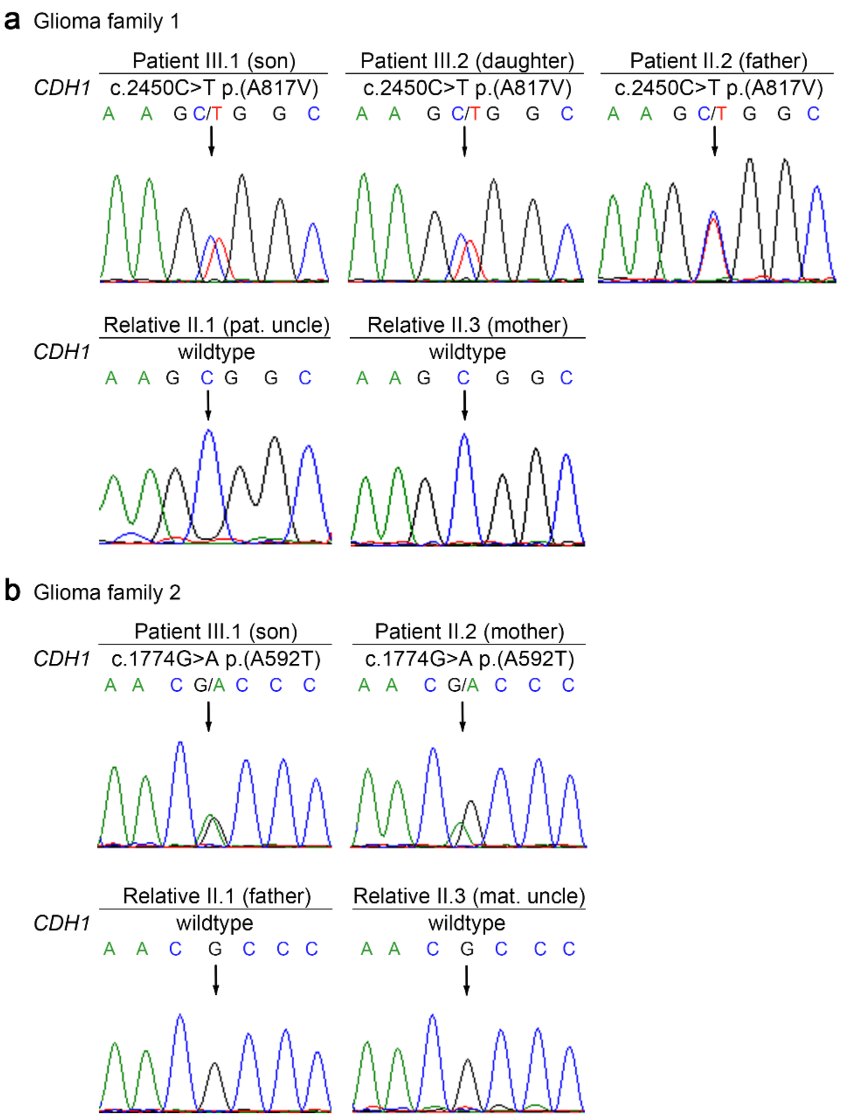

Suppl. Fig. 1 The identified rare heterozygous *CDH1* variants co-segregate with the tumor phenotype in glioma families 1 and 2 as determined by direct sequencing. **a** Electropherograms of glioma family 1 confirming the presence of the heterozygous *CDH1*:c.2450C>T p.(A817V) variant, which was initially identified by whole-genome sequencing, in the germline of patients III.1 (son), III.2 (daughter), and II.2 (father) with oligodendroglioma, WHO grade II/III, IDH-mutant and 1p/19q-codeleted, but not in their tumor-unaffected relatives II.1 (paternal uncle) and II.3 (mother). **b** Electropherograms of glioma family 2 demonstrating the heterozygous *CDH1*:c.1774G>A p.(A592T) variant in the germline of patient III.1 (son) with oligodendroglioma, WHO grade II, IDH-mutant and 1p/19q-codeleted and patient II.2 (mother) with a serous ovarian carcinoma, but not in their tumor-unaffected relatives II.1 (father) and II.3 (maternal uncle). Nucleotide numbering according to nucleotide position in the coding sequence of NCBI Reference Sequence: NM_004360.

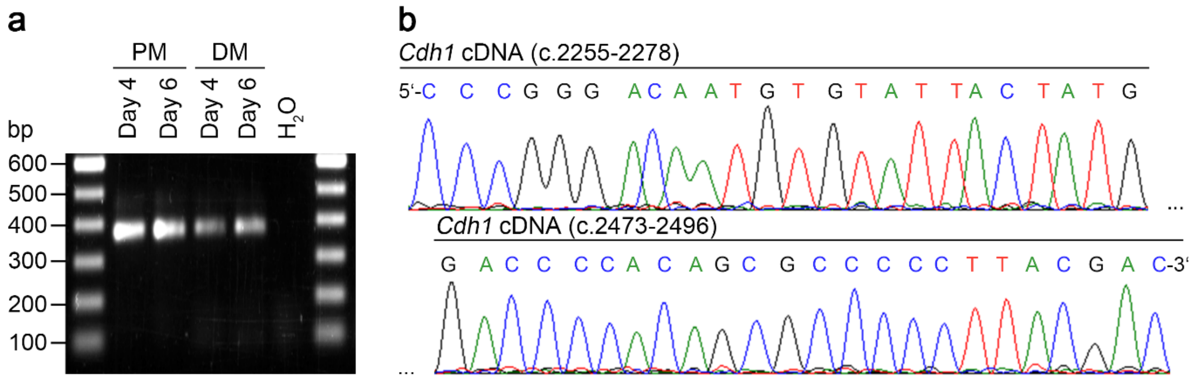

Suppl. Fig. 2 Qualitative *Cdh1* mRNA analysis confirming the presence of *Cdh1*-specific sequences in RNA samples from rat oligodendroglial cultures. Oligodendroglial cells were isolated from neonatal Sprague-Dawley rats (P0-P3) and cultured in either proliferation medium (PM) or differentiation medium (DM) for four or six days. After first-strand cDNA synthesis from total RNA extracted from oligodendroglial cultures at day 4 or day 6, specific oligonucleotides (Suppl. Table 9) were used to amplify a part of the rat *Cdh1* cDNA sequence (NM_031334, c.2165-2536). **a** By gel electrophoresis, a PCR product consistent with the expected rat *Cdh1* cDNA band of 372 bp was detected irrespective of medium used on oligodendroglial cultures or culture duration. **b** Each PCR product shown in (**a**) was subjected to direct sequencing. All obtained electropherograms confirmed the presence of sequences specific for rat *Cdh1* cDNA that are not conserved in other cadherin family members. One of the electropherograms is partly shown.

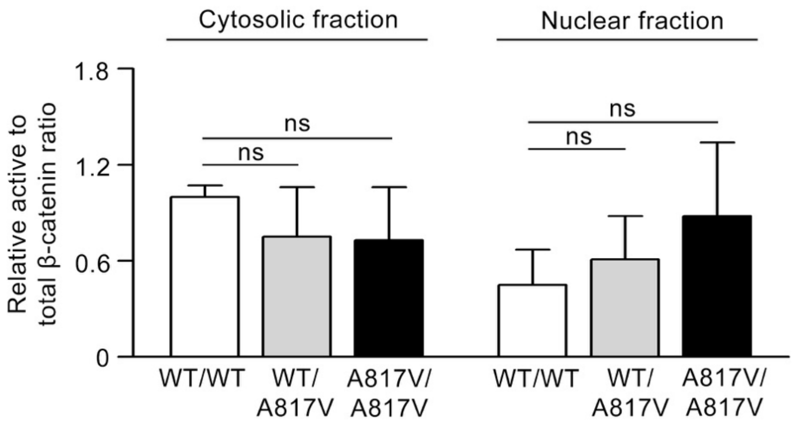

Suppl. Fig. 3 The ratios of active and total β-catenin levels were not significantly different in the cytosolic or nuclear fraction of WT/A817V and A817V/A817V compared to WT/WT cells. Protein bands of active β-catenin (detected using a non-phospho-Ser45-specific antibody) and total β-catenin detected by Western blot analysis after subcellular fractionation (as shown in Fig. 3I) were quantified by densitometry (mean±SD of two independent experiments); ns, not significant (Student's *t* test).

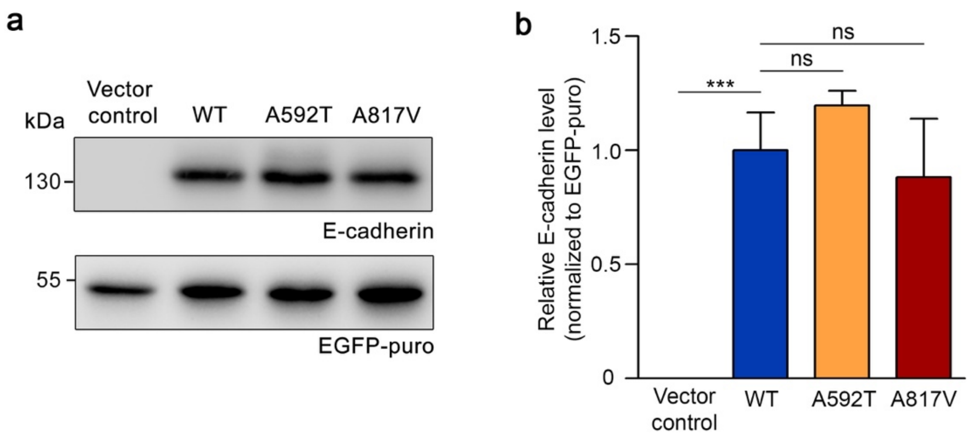

Suppl. Fig. 4 E-cadherin expression in CHO cells transfected with pIRES-EGFP-puro (vector control), pIRES-CDH1 wildtype-EGFP-puro (WT), pIRES-CDH1 A592T-EGFP-puro (A592T), or pIRES-CDH1 A817V-EGFP-puro (A817V), selected by puromycin treatment followed by cell sorting. **a** Cell lysates were examined by Western blot analysis using anti-E-cadherin and anti-GFP antibodies. **b** By densitometric quantification of Western blot analyses (**a**), E-cadherin levels normalized to the co-expressed EGFP-puro were comparable in stably WT and mutant (A592T and A817V) E-cadherin expressing CHO cells (mean±SD of four independent experiments). ***$p \leq 0.001$; ns, not significant (Student's *t* test).

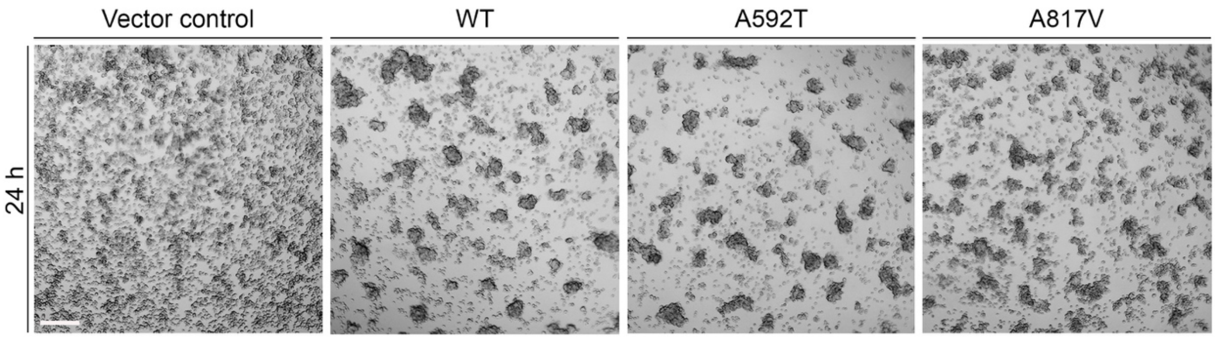

Suppl. Fig. 5 Reduced cell aggregation was observed in CHO cells expressing E-cadherin A817V compared to wildtype E-cadherin expressing cells. Representative images of one of three independent experiments performed in triplicate are shown. Scale bar 200 µm.

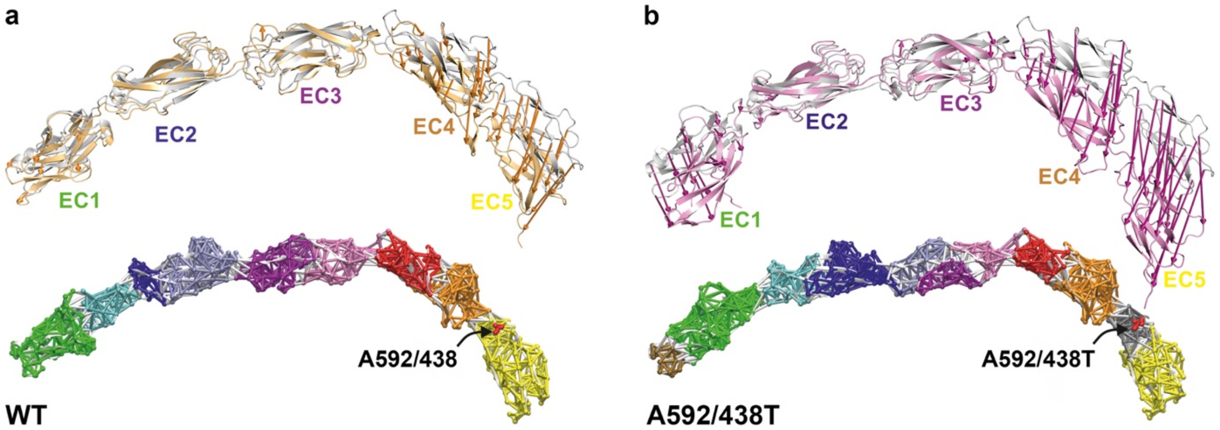

Suppl. Fig. 6 Molecular dynamics (MD) simulations of the mature ectodomain, comprising extracellular cadherin (EC) domains EC1 to EC5, of murine E-cadherin WT (82% sequence identity to the human E-cadherin ectodomain, a crystal structure of the human ectodomain is only available of EC1 and EC2) and E-cadherin A592/438T (the human residue A592 in the pre-protein corresponds to A438 in the mature protein, which is conserved in mouse). **a, b** Upper panel: conformational changes of the E-cadherin WT (**a**) and E-cadherin A592/438T (**b**) ectodomain along the 100 ns MD simulations. Structural changes from the starting structure (grey) are indicated by vectors (**a,** orange; **b,** pink). E-cadherin A592/438T showed increased flexibility particularly of EC1, EC4, and EC5 as illustrated by the larger vector arrows (**b**). Lower panel: dynamical network analysis shows network communities that correlate with the domain architecture of the ectodomain. Network communities of correlated motions are colored individually. Red spheres are used to highlight residue A592/438. The A592/438T variant led to a new network community (grey) around the site of the variant (shown as red spheres) that is uncoupled from the network community (yellow) of the remaining EC5 domain, and also altered the network communities of the EC1 domain (**b**).

SUPPLEMENTARY TABLES

Suppl. Table 1 Analysis of WGS germline data from two siblings (III.1, III.2) and their father (II.2) with oligodendroglioma, WHO grade II/III, IDH-mutant and 1p/19q-codeleted, and their unaffected relatives (II.1, II.3) of glioma family 1 using a linkage-based strategy to identify glioma-predisposing genes

Filtering steps	Number of variants
Total variants in genomes obtained from whole blood	Patient III.1: 4,589,862<br>Patient III.2: 4,704,752<br>Patient II.2: 4,534,339<br>Relative II.1: 4,577,452<br>Relative II.3: 4,672,720
Linkage-based strategy: high quality variants (read depth ≥10, call quality ≥50, allele fraction ≥20% in all samples) shared by patients III.1, III.2 and II.2 affected by oligodendroglioma, WHO grade II/III, IDH-mutant and 1p/19q-codeleted but not found in unaffected relatives (II.1 and II.3) are retained	111,708
Rare variants (≤0.5% in the 1000 Genomes Project, ExAC and gnomAD datasets, and the NHLBI ESP exomes)[a] are retained	9,309
Non-silent (i.e. splice site: up to 2 bases into intron, frameshift, in-frame indels, stop gained/lost and non-synonymous missense) variants and promoter region variants are retained	290
Variants predicted to be deleterious by at least one prediction tool (i.e. SIFT, PolyPhen-2 or RegulationSpotter)[b], and, in the case of non-silent variants, verified by Sanger sequencing to co-segregate with the oligodendroglioma phenotype are retained	30[c]
Variant in cancer predisposing gene (Rahman 2014) is retained	1 (*CDH1*)

[a]1000 Genomes Project data (http://www.internationalgenome.org/), Exome Aggregation Consortium (ExAC) and Genome Aggregation Database (gnomAD) (http://gnomad.broadinstitute.org/), NHLBI Exome Sequencing Project (http://evs.gs.washington.edu/EVS/)

[b]SIFT (http://sift.jcvi.org/), PolyPhen-2 (http://genetics.bwh.harvard.edu/pph2/), RegulationSpotter (http://www.regulationspotter.org/)

[c]Variants are summarized in Suppl. Table 2

Suppl. Table 2 Rare non-silent or promoter region variants predicted to be deleterious that are shared by patients III.1, III.2, and II.2 with oligodendroglioma, WHO grade II/III, IDH-mutant and 1p/19q-codeleted, but not found in tumor-unaffected family members II.1 and II.3, and, thus, co-segregate with the oligodendroglioma phenotype in glioma family 1 (n=30)

Genomic position (GRCh37/ hg19)	Variation type	Gene symbol	Transcript ID	Nucleotide alteration	Deduced protein change	dbSNP[a]	MAF (%)[b]	Prediction according to			Cancer predis-posing gene[f]
								SIFT[c]	PolyPhen-2[d]	Regulation Spotter[e]	
2:109087055-109087056	Deletion	*GCC2*	NM_181453.4	c.1273_1274 delAG	p.E425Sfs*5	rs1477651601	0.001	NA	NA	NA	No
2:183387027	SNV	*PDE1A*	NM_005019.5	c.77A>G	p.K26R	rs144085392	0.124	Damaging	Benign	NA	No
2:186604144	SNV in promoter	*FSIP2-AS2*	NR_110214.1	n.-324C>A	-	rs768058514	0.026	NA	NA	Disease causing	No
2:33701653	SNV in promoter	*RASGRP3*	NM_001139488.2	c.-382T>A	-	-	-	NA	NA	Disease causing	No
2:88383996	SNV	*SMYD1*	NM_198274.4	c.299C>A	p.P100H	rs145518325	0.101	Damaging	Probably damaging	NA	No
2:88383996	SNV in promoter	*MIR4780*	NR_039940.1	n.-1878G>T	-	rs145518325	0.101	NA	NA	Disease causing	No
3:11685869	SNV in promoter	*VGLL4*	NM_001128219.3	c.-877A>G	-	rs182748059	0.013	NA	NA	Disease causing	No
4:177605121	SNV	*VEGFC*	NM_005429.5	c.1219C>T	p.R407C	rs373412644	0.003	Damaging	Probably damaging	NA	No
4:36307915-36307917	Deletion	*DTHD1*	NM_001170700.3	c.1374_1376 delTGT	p.V459del	rs747697241	0.029	NA	NA	NA	No
4:87662802	SNV	*PTPN13*	NM_080685.2	c.2320A>G	p.T774A	rs776072423	0.003	Tolerated	Possibly damaging	NA	No
4:87855771	SNV in promoter	*AFF1*	NM_001166693.2	c.-658T>A	-	rs542606613	0.233	NA	NA	Disease causing	No
6:97677161-97677163	Deletion	*MMS22L*	NM_198468.4	c.1646_1648 delGTC	p.S549_H550 delinsN	rs1165215914	0.001	NA	NA	NA	No
6:99887715	SNV	*USP45*	NM_001080481.2	c.2090G>A	p.R697H	rs139278154	0.002	Damaging	Probably damaging	NA	No
8:70405036	SNV in promoter	*SULF1*	NM_001128206.2	c.-553A>G	-	rs867273836	0.013	NA	NA	Disease causing	No
9:32552878	SNV	*SMIM27*	NM_001349118.1	c.125A>G	p.K42R	rs554234544	0.081	NA	Possibly damaging	NA	No
9:5968303	SNV	*KIAA2026*	NM_001017969.3	c.1928A>G	p.H643R	rs201096994	0.108	Damaging	Possibly damaging	NA	No
9:90535756	SNV	*SPATA31C1*	NM_001145124.1	c.934G>C	p.D312H	rs201907359	0.262	NA	Possibly damaging	NA	No
11:114577269	SNV	*NXPE2*	NM_182495.6	c.1297C>T	p.R433W	rs763575029	0.005	Damaging	Probably damaging	NA	No
11:34643113	Deletion in promoter	*EHF*	NM_001378053.1	c.-845delA	-	-	-	NA	NA	Disease causing	No

11:59211102	SNV	*OR5A1*	NM_001004728.1	c.461G>T	p.G154V	rs765560979	0.001	Tolerated	Probably damaging	NA	No
11:94760049	SNV	*KDM4E*	NM_001161630.1	c.1328G>A	p.R443H	rs374081859	0.031	Tolerated	Possibly damaging	NA	No
15:78556472	SNV in promoter	*DNAJA4*	NM_001130182.1	c.-634G>C	-	rs939059379	0.006	NA	NA	Disease causing	No
16:23569332	SNV in promoter	*EARS2*	NR_003501.1	n.-636C>A	-	rs375139253	0.170	NA	NA	Disease causing	No
16:31092320	SNV	*ZNF646*	NM_014699.4	c.4675T>C	p.C1559R	-	-	Damaging	Probably damaging	NA	No
16:68867203	SNV	*CDH1*	NM_004360.5	c.2450C>T	p.A817V	rs587782024	0.003	Damaging	Probably damaging	NA	Yes
16:69184603	SNV	*UTP4*	NM_032830.3	c.902T>C	p.I301T	-	-	Damaging	Possibly damaging	NA	No
17:45266575-45266576	Insertion in promoter	*CDC27*	ENST00000531206.5	c.-38_-37 insGGGGTG	-	-	-	NA	NA	Disease causing	No
19:11332545	SNV	*DOCK6*	NM_020812.4	c.3532C>T	p.R1178W	rs375012919	0.009	Damaging	Possibly damaging	NA	No
19:9086143	SNV	*MUC16*	NM_024690.2	c.5672G>A	p.S1891N	rs138381516	0.412	Damaging	Possibly damaging	NA	No
20:48522388	SNV	*SPATA2*	NM_001135773.1	c.1331C>T	p.P444L	rs143193219	0.001	Damaging	Benign	NA	No

[a]SNP database ID (http://www.ncbi.nlm.nih.gov/SNP/)
[b]According to the gnomAD browser v2.1.1 (all samples, http://gnomad.broadinstitute.org/)
[c]SIFT (http://sift.jcvi.org/)
[d]PolyPhen-2 (http://genetics.bwh.harvard.edu/pph2/)
[e]RegulationSpotter (http://www.regulationspotter.org/)
[f]According to Rahman 2014

Suppl. Table 3 Oligonucleotides used for amplification and sequencing of human *CDH1* (NM_004360) coding exons

Exon	Primer sequence (5' to 3')	
	Forward	Reverse
1	GAACCCTCAGCCAATCAGC	GTGACGACGGGAGAGGAAG
2	AGTCACCCGGTTCCATCTAC	CCTTTCCAACCCCTCCCTAC
3	TTGGAGAAGGAATGCTCTTGT	CAGCGCACTAAAACAACAGC
4	GCTGTCTGGCTAGGTTGGA	GCCAAATCCCTCCCAGAGAA
5	AGGGAAAAGACCCAGTGTTGG	GATGTTACCCCGGTGTCAAC
6	GAGCTCAAGTCACCCTCACT	CTTTGGGCTTGGACAACACT
7	ATTAGGGAACTCTGACACGGT	TTGCTTTGTCCACGGGATTG
8	TGGTCCTGACTTGGTTGTGT	AGACTTCGCCCATGAGCAGT
9	AATCCTTTAGCCCCCTGAGACT	AAGATACCAGGGGACAAGGGTAT
10	GAAGCCATGGTAAGTAATTGTGG	TGAAAGGAGCACAGATAAAGGGA
11	ACGTTGGAAGTAACCATATAACTG	TACCACTACACATCTATGCATGT
12	ATTACTGTTGCCAAGCTGCC	CATGGCAGTTGGAGCAAAGT
13	TCCTCCCCTGGTCTCATCAT	GCTGGCATAACTTGGGAGT
14	GGCTCTCAACACTTGCTCTG	TCAGAGCTGTTTCAAATGCCT
15	ACATAGCCCTGTGTGTATGACT	GCTCAGGCAAGCTGAAAACA
16	TTCTTGCCCCAGATGACAGG	GTGATTTCTGCATTTCCCAGC

Suppl. Table 4 Oligonucleotides used for amplification and sequencing of human *POT1* (NM_015450) coding exons

Exon	Primer sequence (5' to 3')	
	Forward	Reverse
1	GACATTCTGTTCAGTCAACCACAG	CATGAGGATGGTGTAAACCAGTC
2	CTACAGGAGCTTCGAATCTACCAAA	TAACTCCTGTCTGCTTCTCCCAAA
3	CAACCCCGAACCGTGTTAACTATAAT	GAAACTTACTCCAATGTTTCACACACG
4	GCTAGGGAAAGTTGATCTAGTTATCTG	GCAATGGTCTAGATATGAGAGTAAAGGG
5	TAGCATTTTCCTGGTCCATGAAGTG	GTCAGACCAAGTAGTTTATCTGCAG
6	ATCCTGAGAACTGTCCTGAGAAC	ATCAGCAGATATTCCAGACAACAG
7	GCTGTGTGCATTGCCTTATTTCAG	TGGTTTCACGTTGCTGTTTGGC
8	GCAGTGTGTATTGAAAGCCAAG	TCTGGGGAATGAAAGCAGTG
9	CTTTGGTTCGTAGGTTGTGCATC	CACAGCATGCTTTATCTCATCAGA
10	ATAGTGCCAATATTCAGAGGCATAAG	CTAGACTGTATCACCTATACATGTAACC
11	AGCTGATATTCAACCACACTCG	CTGCTACTACTTAGCTCTATGAGCC
12	CCCGGAAAATCTGATGAAAGCAATAG	GTTTACTTTTTGGCATAGGCCACAG
13	ATCATGAATGTGGCATATCTCACC	GCCAACAAGACACGGTAGAAGAA

14	ATTTGGTTCAGGAGGATGCATG	GCCTTTATCAGAGTGACTAAGCTG
15	GTTATTTGTTCTGCAAAGTGAATTAT CTCCA	GAACGTGTGCTTATATATATTTGAACC TAGT
16	CCAAAGTCTCAGCATTTTTGTGC	CATGTTCAGCACATGACCCCA
17	TAGGGGATGCAGTATTCTTCTG	CGGTGCCTGCTTGAAGAAATA
18	CCTCCAGTTCAATTCTGACACTATC	TAAGTCCTTTTAGGAACAAAGCAGG
19	GGAGTTGAGACCAGCATTCTAGTT	TTTTATCCCTGCCACTCTCTTCATC
20	GATGTACTCACTGTTACCTGCTTGT	GCCATTATTTACCTTGCACCCAG

Suppl. Table 5 Oligonucleotides used to subclone the human *CDH1* open reading frame amplified from hE-cadherin-pcDNA3 (#45769; Addgene) into the *Xho*I-linearized pIRES-EGFP-puro vector (#45567; Addgene)

**Restriction site**	**Primer sequence (5' to 3')**	
	**Forward**	**Reverse**
*Xho*I	GGACTCAGATCTCGAGATGGGCC CTTGGAGCCGC	GAAGCTTGAGCTCGAGCTAGTCGTCC TCGCCGCC

Suppl. Table 6 Oligonucleotides used for site-directed mutagenesis of human *CDH1* variants into a pIRES-CDH1 wildtype-EGFP-puro construct

**Variant**	**Primer sequence (5' to 3')**	
	**Forward**	**Reverse**
c.1774G>A p.(A592T)	GACAACACCCCCATACCAGAAC CTC	ATTCACATCAGACAGGATCAGCA GAAG
c.2450C>T p.(A817V)	ATGAAAATCTGAAAGTGGCTGA TACGGATCCCACAGCCCCGCCT	CAATAAAATTTCCAATTTCATCGG GATTGGCAGGGCGGGGAA

Suppl. Table 7 Oligonucleotides used for quantitative PCR of rat *Cdh1* cDNA (NM_031334)

**Primer sequence (5' to 3')**	
**Forward**	**Reverse**
GGCCCTCCTGATTCTGATCC	CGGGTATCGTCATCTGGTGG

Suppl. Table 8 Oligonucleotides used for quantitative PCR of rat *Pgk1* cDNA (NM_053291), as described in Langnaese et al. 2008

**Primer sequence (5' to 3')**	
**Forward**	**Reverse**
ATGCAAAGACTGGCCAAGCTAC	AGCCACAGCCTCAGCATATTTC

Suppl. Table 9 Oligonucleotides used for amplification and sequencing of rat *Cdh1* cDNA (NM_031334)

**Primer sequence (5' to 3')**	
**Forward**	**Reverse**
TGGCCCTCCTGATTCTGATC	CTTCAGAACCACTCCCCTCA

Suppl. Table 10 Oligonucleotides used for CRISPR knock-in of the human *CDH1* A817V variant in HEK293T cells

**Primer name**	**Primer sequence (5' to 3')**
gRNA-*CDH1*-FP (sense)	AAAC**GCCTTATGATTCTCTGCTCG**C
gRNA-*CDH1*-RP (antisense)	CACCG**CGAGCAGAGAATCATAAGGC**
ssODN-*CDH1*-A817V	TCGTACCTTACATATTGCTAGACTTCTTGCCCCAGATGA CAGGTGTGCCCTTCCTTTCACTAAAAGATGCTTTTGTCC CTTCTTCTTTAGAATCTGAAAG***T***GGCTGATACTGACCC CACAGC***T***CCGCCTTATGATTCTCTGCTCGTGTTTGACT ATGAAGGAAGCGGTTCCGAAGCTGCTAGTCTGAGCTCC CTGAACTC

Given are sense and antisense oligonucleotides designed for gRNA (target sequence in bold print) insertion into the pSpCas9(BB)-2A-GFP vector (#48138, Addgene), and sequence of the synthetic single-stranded oligo DNA nucleotide (ssODN) containing the *CDH1*:c.2450C>T p.(A817V) variant and the synonymous PAM variant c.2472C>T (both indicated in bold print) used as homology-directed repair template in the CRISPR/Cas9 protocol.

Suppl. Table 11 Oligonucleotides used to determine the sequence of gRNA on- and off-target sites in the three HEK293T cell clones selected for further analysis harboring either human wildtype *CDH1*, a heterozygous or homozygous *CDH1* A817V knock-in

**Locus**	**Primer sequence (5' to 3')**	
	**Forward**	**Reverse**
CDH1-E16 on-target	TCTGGGTGCATTGTCGTACC	TCTCAAGGGAAGGGAGCTGA
PARP9-E7 off-target	CCGTGTTCTAATAGAACTGAG GTCCTC	TGATCAGGGTAGAGAGTAGGCAGGTA
AC073218.1 off-target	CAACCAAATAGGTGGATCCTG CTCT	ACTAGTCAGTTCACAAATTGTTGTGAG AG
VAMP1-E3 off-target	GTGGAGGAGGTAGGTAGATA GCTGT	GAGGAGGGGACAAGAAAATTCACC
VANGL1-E7 off-target	GACCAGAAGTGTGGTGGAAC CC	ACATTCCTCTAAACATGTGTCTGCCTA C
STRN4-E26 off-target	CAAGGTCTTCGTATGATGCCC ACC	CTCTGCACCTCCAGCGAGG

E, exon

Suppl. Table 12 Rare non-silent *CDH1* variants predicted to be deleterious identified in leukocyte DNA of glioma families or tumor DNA of 99 oligodendrogliomas, WHO grade II/III, IDH-mutant and 1p/19q-codeleted and their allele frequency compared to controls

Exon	Genomic position (GRCh37/hg19)	Nucleotide alteration	Deduced protein change	Protein domain	dbSNP[a]	Prediction according to		Minor allele frequency (allele count/allele number)		
						SIFT[b]	PolyPhen-2[c]	Glioma families[d]	Oligodendrogliomas[e]	Controls[f]
12	16:68855966	c.1774G>A	p.A592T	Ectodomain	rs35187787	Damaging	Benign	0.03333 (1/30)	0.01333 (2/150)[g,h]	0.00297 (357/120276)
12	16:68856106	c.1913G>A	p.W638*	Ectodomain	-	NA	NA	-	**0.00667** (1/150)[g]	0.00000 (0/120292)
14	16:68862157	c.2245C>T	p.R749W	Intracellular	rs776975632	Damaging	Probably damaging	-	**0.00510** (1/192)[h]	0.00000 (0/120292)
16	16:68867203	c.2450C>T	p.A817V	Intracellular	rs587782024	Damaging	Probably damaging	**0.03333** (1/30)	-	0.00003 (3/109404)
16	16:68867221	c.2468C>T	p.T823I	Intracellular	-	Damaging	Probably damaging	-	**0.01064** (2[h,i]/186)	0.00000 (0/120292)
16	16:68867343	c.2590G>A	p.E864K	Intracellular	rs142927667	Damaging	Possibly damaging	-	**0.00532** (1/186)[h]	0.00003 (3/109408)

Given are all identified rare (minor allele frequency, MAF ≤0.5%), non-silent (i.e. splice site, frameshift, in-frame indels, stop gained/lost and non-synonymous missense) variants predicted to be deleterious by at least one prediction tool, i.e. SIFT or PolyPhen-2, in the *CDH1* gene (NM_004360) and their allele frequencies in glioma families (leukocyte DNA) and oligodendrogliomas, WHO grade II/III, IDH-mutant and 1p/19q-codeleted (tumor DNA) compared to controls. The MAFs indicated in bold print significantly differ from controls ($p \leq 0.01$, two-sided Fisher's exact test).

[a]SNP database ID (http://www.ncbi.nlm.nih.gov/SNP/)

[b]SIFT (http://sift.jcvi.org/)

[c]PolyPhen-2 (http://genetics.bwh.harvard.edu/pph2/)

[d]Leukocyte DNA of glioma families with at least two glioma patients each was investigated (n=15)

[e]Tumor DNA of oligodendrogliomas, WHO grade II/III, IDH-mutant and 1p/19q-codeleted was investigated (*CDH1* exons 1-11 n=28-65, exon 12 n=75, exon 13 n=72, exon 14 n=96, exon 15 n=99, exon 16 n=93)

[f]Control individuals according to the gnomAD browser v2.1.1 (http://gnomad.broadinstitute.org/) (n=60,146)

[g]Leukocyte DNA was not available to determine whether the variant is present in the germline or not

[h]Variant not detected in DNA from non-neoplastic tissue or whole blood

[i]Homozygous variant detected in one anaplastic oligodendroglioma, WHO grade III, IDH-wildtype and 1p/19q-codeleted

Suppl. Table 13 Oligodendroglioma patients and oligodendrogliomas harboring rare non-silent *CDH1* variants predicted to be deleterious

Patient		Gender	Age at diagnosis (years)	Glioma histology, WHO grade	Glioma localization	Molecular characteristics	Nucleotide alteration	Deduced protein change	Previously described in the germline of patients diagnosed with: Cancer entity	References
***CDH1* variants identified in the leukocyte DNA of familial glioma patients**										
**Glioma family 1**	**III.1**	Male	37	O II	Left frontal	IDH-mutant, 1p/19q-codeleted	c.2450C>T	p.A817V	Colorectal cancer	Raskin et al. 2017
**Glioma family 1**	**III.2**	Female	38	AO III	Left frontal	IDH-mutant, 1p/19q-codeleted	c.2450C>T	p.A817V	Colorectal cancer	Raskin et al. 2017
**Glioma family 1**	**II.2**	Male	i) 51<br>ii) 53<br>iii) 70	i) A II<br>ii) and iii) AO III	Left frontal	i) Not evaluated<br>ii) and iii) IDH-mutant, 1p/19q-codeleted	c.2450C>T	p.A817V	Colorectal cancer	Raskin et al. 2017
**Glioma family 2**	**III.1**	Male	30	O II	Right frontal paramedian	IDH-mutant, 1p/19q-codeleted	c.1774G>A	p.A592T	i) Prostate cancer<br>ii) Mixed gastric cancer<br>iii) Invasive lobular breast cancer<br>iv) Colon and colorectal cancer	i) Jonsson et al. 2002<br>ii) Garziera et al. 2013<br>iii) Valente et al. 2014<br>iv) Salashor et al. 2001
***CDH1* variants identified in the tumor DNA of oligodendrogliomas**										
**O11**		Female	76	AO III	Right frontal	IDH-wildtype, 1p/19q-codeleted	c.2468C>T	p.T823I	-	-
**O12**		Female	43	AO III	Left frontal	IDH-mutant, 1p/19q-codeleted	c.2590G>A	p.E864K	i) Colon cancer (Lynch syndrome)<br>iii) Breast and/or ovarian cancer	i) Yurgelun et al. 2015<br>ii) Singh et al. 2018
**O40**		Female	33	AO III	Occipital lobe	IDH-mutant, 1p/19q-codeleted	c.2245C>T	p.R749W	Diffuse gastric cancer	Kaurah et al. 2007
**O46**		Male	36	O II	Right frontal	IDH-mutant, 1p/19q-codeleted	c.1774G>A	p.A592T	i) Prostate cancer<br>ii) Mixed gastric cancer<br>iii) Invasive lobular breast cancer<br>iv) Colon and colorectal cancer	i) Jonsson et al. 2002<br>ii) Garziera et al. 2013<br>iii) Valente et al. 2014<br>iv) Salashor et al. 2001
**O88**		Male	58	AO III	Right frontal parasagittal	IDH-mutant, 1p/19q-codeleted	c.1774G>A	p.A592T	i) Prostate cancer<br>ii) Mixed gastric cancer<br>iii) Invasive lobular breast cancer<br>iv) Colon and colorectal cancer	i) Jonsson et al. 2002<br>ii) Garziera et al. 2013<br>iii) Valente et al. 2014<br>iv) Salashor et al. 2001
**O47**		Male	38	AO III	-	IDH-mutant, 1p/19q-codeleted	c.1913G>A	p.W638*	Diffuse gastric cancer	Kaurah et al. 2007

NCBI Reference Sequence: NM_004360

O II, oligodendroglioma, WHO grade II; AO III, anaplastic oligodendroglioma, WHO grade III; A II, astrocytoma

Suppl. Table 14 Rare non-silent variant predicted to be deleterious identified by mutational analysis of *CDH1* exons 14 to 16 in tumor DNA of 65 renal cell carcinomas (RCC) and its allele frequency compared to controls

**Exon**	**Genomic position (GRCh37/hg19)**	**Nucleotide alteration**	**Deduced protein change**	**Protein domain**	**dbSNP[a]**	**Prediction according to**		**Minor allele frequency (allele count/allele number)**			
						**SIFT[b]**	**PolyPhen-2[c]**	**Chromophobe RCC (n=26)**	**Clear cell RCC (n=21)**	**Papillary RCC (n=18)**	**Controls[d] (n=60,146)**
16	16:68867310	c.2557T>C	p.S853P	Intracellular	rs765978401	Damaging	Probably damaging	**0.03846** (2[e,f]/52)	-	-	0.00000 (0/120292)

Given are all rare (minor allele frequency, MAF ≤0.5%), non-silent (i.e. splice site, frameshift, in-frame indels, stop gained/lost and non-synonymous missense) variants predicted to be deleterious by at least one prediction tool, i.e. SIFT or PolyPhen-2, in exons 14 to 16 of the *CDH1* gene (NM_004360) and their allele frequency in RCCs (tumor DNA) compared to controls. The MAF indicated in bold print significantly differs from controls (p ≤0.001, two-sided Fisher's exact test).

[a]SNP database ID (http://www.ncbi.nlm.nih.gov/SNP/)

[b]SIFT (http://sift.jcvi.org/)

[c]PolyPhen-2 (http://genetics.bwh.harvard.edu/pph2/)

[d]Control individuals according to the gnomAD browser v2.1.1 (http://gnomad.broadinstitute.org/)

[e]Homozygous variant in one chromophobe RCC

[f]Leukocyte DNA was not available to determine whether the variant is present in the germline or not

Suppl. Table 15 No indels at off-target sites were detected in the selected *CDH1*$^{WT/WT}$, *CDH1*$^{WT/A817V}$, and *CDH1*$^{A817V/A817V}$ HEK293T single cell clones

Off-target genes / lincRNA	Mismatches at off-target sites and CFD score		Results from sequence analysis of genomic DNA amplicons from off-target sites		
	Compared to gRNA on-target site in *CDH1* (exon 16): GCCTTATGATTCTCTGCTCG	CFD score	*CDH1*$^{WT/WT}$ HEK293T clone	*CDH1*$^{WT/A817V}$ HEK293T knock-in clone	*CDH1*$^{A817V/A817V}$ HEK293T knock-in clone
PARP9 (exon 7)	TG.......C.........T	0.1457	No indel	No indel	No indel
AC073218.1 (lincRNA)	.......TT....A.T....	0.1005	No indel	No indel	No indel
VAMP1 (exon 3)	.A...G..........G.T.	0.0814	No indel	No indel	No indel
VANGL1 (exon 7)	...A....C..GG.......	0.0154	No indel	No indel	No indel
STRN4 (exon 26)	..A..C...G.......A..	0.0094	No indel	No indel	No indel

All coding off-target sites with homology to the given guide RNA (gRNA) on-target site in *CDH1* exon 16 were determined by the CRISPOR webtool (http://crispor.tefor.net/), and are sorted by their cutting frequency determination (CFD) score. Sequence analysis of genomic DNA amplicons was performed by direct sequencing (oligonucleotide sequences are given in Suppl. Table 11).

References

Darden T, York D, Pedersen L (1993) Particle Mesh Ewald - an Nlog(N) method for Ewald Sums in Large Systems. J Chem Phys 98:10089-10092 Doi 10.1063/1.464397

Garziera M, Canzonieri V, Cannizzaro R, Geremia S, Caggiari L, De Zorzi M, Maiero S, Orzes E, Perin T, Zanussi S, De Paoli P, De Re V (2013) Identification and characterization of *CDH1* germline variants in sporadic gastric cancer patients and in individuals at risk of gastric cancer. PLoS One 8:e77035 Doi 10.1371/journal.pone.0077035

Humphrey W, Dalke A, Schulten K (1996) VMD: Visual molecular dynamics. J Mol Graph Model 14:33-38 Doi 10.1016/0263-7855(96)00018-5

Jonsson BA, Bergh A, Stattin P, Emmanuelsson M, Grönberg H (2002) Germline mutations in E-cadherin do not explain association of hereditary prostate cancer, gastric cancer and breast cancer. Int J Cancer 98:838-843 Doi 10.1002/ijc.10258

Jorgensen WL, Chandrasekhar J, Madura JD, Impey RW, Klein ML (1983) Comparison of Simple Potential Functions for Simulating Liquid Water. J Chem Phys 79:926-935 Doi 10.1063/1.445869

Kaurah P, MacMillan A, Boyd N, Senz J, De Luca A, Chun N, Suriano G, Zaor S, Van Manen L, Gilpin C, Nikkel S, Connolly-Wilson M, Weissman S, Rubinstein WS, Sebold C, Greenstein R, Stroop J, Yim D, Panzini B, McKinnon W, Greenblatt M, Wirtzfeld D, Fontaine D, Coit D, Yoon S, Chung D, Lauwers G, Pizzuti A, Vaccaro C, Redal MA, Oliveira C, Tischkowitz M, Olschwang S, Gallinger S, Lynch H, Green J, Ford J, Pharoah P, Fernandez B, Huntsman D (2007) Founder and recurrent *CDH1* mutations in families with hereditary diffuse gastric cancer. JAMA 297:2360-2372 Doi 10.1001/jama.297.21.2360

Langnaese K, John R, Schweizer H, Ebmeyer U, Keilhoff G (2008) Selection of reference genes for quantitative real-time PCR in a rat asphyxial cardiac arrest model. BMC Mol Biol 9:53 Doi 10.1186/1471-2199-9-53

Rahman N (2014) Realizing the promise of cancer predisposition genes. Nature 505:302-308 Doi 10.1038/nature12981

Raskin L, Guo Y, Du L, Clendenning M, Rosty C, Lindor NM, Gruber SB, Buchanan DD (2017) Targeted sequencing of established and candidate colorectal cancer genes in the Colon Cancer Family Registry Cohort. Oncotarget 8:93450-93463 Doi 10.18632/oncotarget.18596

Salahshor S, Hou H, Diep CB, Loukola A, Zhang H, Liu T, Chen J, Iselius L, Rubio C, Lothe RA, Aaltonen L, Sun XF, Linmark G, Lindblom A (2001) A germline E-cadherin mutation in a family with gastric and colon cancer. Int J Mol Med 8:439-443 Doi 10.3892/ijmm.8.4.439

Sethi A, Eargle J, Black AA, Luthey-Schulten Z (2009) Dynamical networks in tRNA: protein complexes. P Natl Acad Sci USA 106:6620-6625 Doi 10.1073/pnas.0810961106

Singh J, Thota N, Singh S, Padhi S, Mohan P, Deshwal S, Sur S, Ghosh M, Agarwal A, Sarin R, Ahmed R, Almel S, Chakraborti B, Raina V, DadiReddy PK, Smruti BK, Rajappa S, Dodagoudar C, Aggarwal S, Singhal M, Joshi A, Kumar R, Kumar A, Mishra DK, Arora N, Karaba A, Sankaran S, Katragadda S, Ghosh A, Veeramachaneni V, Ramesh H, Mannan AU (2018) Screening of over 1000 Indian patients with breast and/or ovarian cancer with a multi-gene panel: prevalence of *BRCA1/2* and non-*BRCA* mutations. Breast Cancer Res Treat 170:189-196 Doi 10.1007/s10549-018-4726-x

Valente AL, Rummel S, Shriver CD, Ellsworth RE (2014) Sequence-based detection of mutations in cadherin 1 to determine the prevalence of germline mutations in patients with invasive lobular carcinoma of the breast. Hered Cancer Clin Pract 12:17 Doi 10.1186/1897-4287-12-17

Yurgelun MB, Allen B, Kaldate RR, Bowles KR, Judkins T, Kaushik P, Roa BB, Wenstrup RJ, Hartman AR, Syngal S (2015) Identification of a Variety of Mutations in Cancer Predisposition Genes in Patients With Suspected Lynch Syndrome. Gastroenterology 149:604-613.e620 Doi 10.1053/j.gastro.2015.05.006
